# HisCMCL: cross-modal contrastive learning with hierarchical multi-scale fusion for spatial expression prediction

**DOI:** 10.1093/bioinformatics/btag342

**Published:** 2026-05-29

**Authors:** Chengju Liu, Fangfang Zhu, Wenwen Min

**Affiliations:** School of Information Science and Engineering, Yunnan University, Kunming, Yunnan 650500, China; School of Health and Nursing, Yunnan Open University, Kunming, Yunnan 650500, China; School of Information Science and Engineering, Yunnan University, Kunming, Yunnan 650500, China

## Abstract

**Motivation:**

High costs and operational complexity limit the clinical application of spatial transcriptomics (ST). Inferring ST from pathology images is a promising alternative, the core of which lies in effectively aligning image and gene expression features. However, existing models are mostly limited to single-scale and single-slice modeling. This not only fails to connect microscopic cells with macroscopic tissues but also restricts generalization due to the inability to extract cross-sample shared features. Furthermore, the inherent representational differences between modalities further exacerbate the difficulty of feature alignment.

**Results:**

To address these challenges, we propose HisCMCL, a multimodal framework. The model combines multi-scale features with a cross-attention mechanism to jointly capture local morphology and global context. Additionally, it fuses spatial location information and utilizes a contrastive learning strategy to facilitate the effective alignment of image and transcriptomic features. Evaluations on four public datasets demonstrate that HisCMCL outperforms existing baseline methods in predictive performance. It exhibits good structural consistency in identifying cancer and immune markers and delineating tumor regions, offering new insights for spatial expression inference.

**Availability and implementation:**

The code of HisCMCL is available at https://github.com/wenwenmin/HisCMCL.

## 1 Introduction

Spatial Transcriptomics (ST) technology provides a new way to study tissue heterogeneity and gene function by mapping gene expression to specific locations in tissue slices (Min *et al.* 2025, Rao *et al.* 2021, Williams *et al.* 2022). Although ST can generate complex gene expression profiles using many spatial spots, its high cost and low throughput limit its wide clinical application (Zhao *et al.* 2021, Min *et al.* 2024, Shi *et al.* 2025b). In contrast, Hematoxylin and Eosin (H&E) stained pathology images are the gold standard for clinical diagnosis (Campanella *et al.* 2019, Dimitriou *et al.* 2019, Waylen *et al.* 2020). They are inexpensive and widely available. Since each ST spot naturally aligns with a corresponding H&E image patch, predicting spatial gene expression directly from pathology images has become a mainstream direction in bioinformatics (Mondol *et al.* 2023, Chen *et al.* 2025, El Nahhas *et al.* 2025).

In recent years, several studies have focused on directly predicting spatial gene expression from pathology images. Early methods primarily adopted an end-to-end regression approach, using image encoders to extract local morphological features to directly predict the gene expression profiles of the corresponding spatial spots (He *et al.* 2020, Jia *et al.* 2024). To overcome the limitations of relying solely on local features, some studies introduced Vision Transformers (Han *et al.* 2022) (ViT) and Graph Neural Networks (Corso *et al.* 2024) (GNN) to capture the inherent spatial dependencies and topological structures within tissue slices (Pang *et al.* 2021, Zeng *et al.* 2022). Furthermore, some works achieved preliminary association between image and gene features by constructing a joint latent space (Khosla *et al.* 2020, Xie *et al.* 2023, Shi *et al.* 2025c). Other methods incorporated biological prior knowledge (Ganguly *et al.* 2025, Huang *et al.* 2025, Shi *et al.* 2025a) as structural constraints to further improve prediction accuracy.

Despite the progress these methods have made in establishing image-to-gene mappings, some limitations remain. First, existing models typically extract only single-scale features, severing the connection between microscopic cellular details and macroscopic tissue architecture. This lack of multi-scale contextual information makes it difficult for models to accurately predict gene expression that highly depends on the surrounding tissue microenvironment. Second, existing paradigms are mostly limited to spatial modeling within a single slice, ignoring potential shared expression patterns across different slices. In fact, the same type of tissue or disease across different individuals shares similar morphological and gene expression patterns, yet also exhibits significant sample heterogeneity due to individual differences and disease progression. This complex sample diversity makes models trained solely on single slices highly prone to overfitting. Furthermore, the inherent differences between pathology images and gene expression make it difficult to effectively align these two types of data, which directly limits inference accuracy (Lin *et al.* 2026). Therefore, synergistically fusing multi-scale image features, effectively extracting shared information across samples, and achieving accurate data alignment are crucial for improving prediction accuracy and enhancing model generalization.

To address these challenges, we propose HisCMCL, a multimodal deep learning framework designed for accurate and generalizable spatial gene expression prediction. To overcome the limitations of single-scale feature extraction, HisCMCL introduces a multi-scale feature extraction strategy and a cross-attention mechanism, enabling the simultaneous capture of microscopic cellular details and macroscopic tissue architecture. This achieves an effective integration of local and global contextual information. Furthermore, to address the issue of poor cross-sample generalization, we designed a robust contrastive learning mechanism that establishes precise modality alignment between image morphology and transcriptomic profiles. This not only enhances the model’s resistance to experimental noise and individual differences but also greatly improves its predictive ability on unseen samples. The main contributions of this paper are summarized as follows:

- We propose HisCMCL, a novel multimodal deep learning framework that establishes accurate mappings between histological images and gene expression while enabling cross-slice prediction, effectively enhancing the robustness and generalizability of spatial gene expression prediction.
- We developed a multi-scale cross-attention mechanism and a spatial-aware contrastive learning strategy. These mechanisms and strategies enable the integration of comprehensive histological features and the alignment of consistent cross-slice representations.
- Evaluated across four public ST datasets spanning diverse tissues and cancer subtypes, HisCMCL sets a new state-of-the-art by outperforming eight baseline models in accuracy and co-expression fidelity. This superior predictive power underpins the framework’s practical utility in identifying cancer-related genes and precisely delineating tumor boundaries, showing high spatial concordance with pathologist-verified annotations.

## 2 Methods

### 2.1 Overview of the proposed HisCMCL

**Figure 1:**
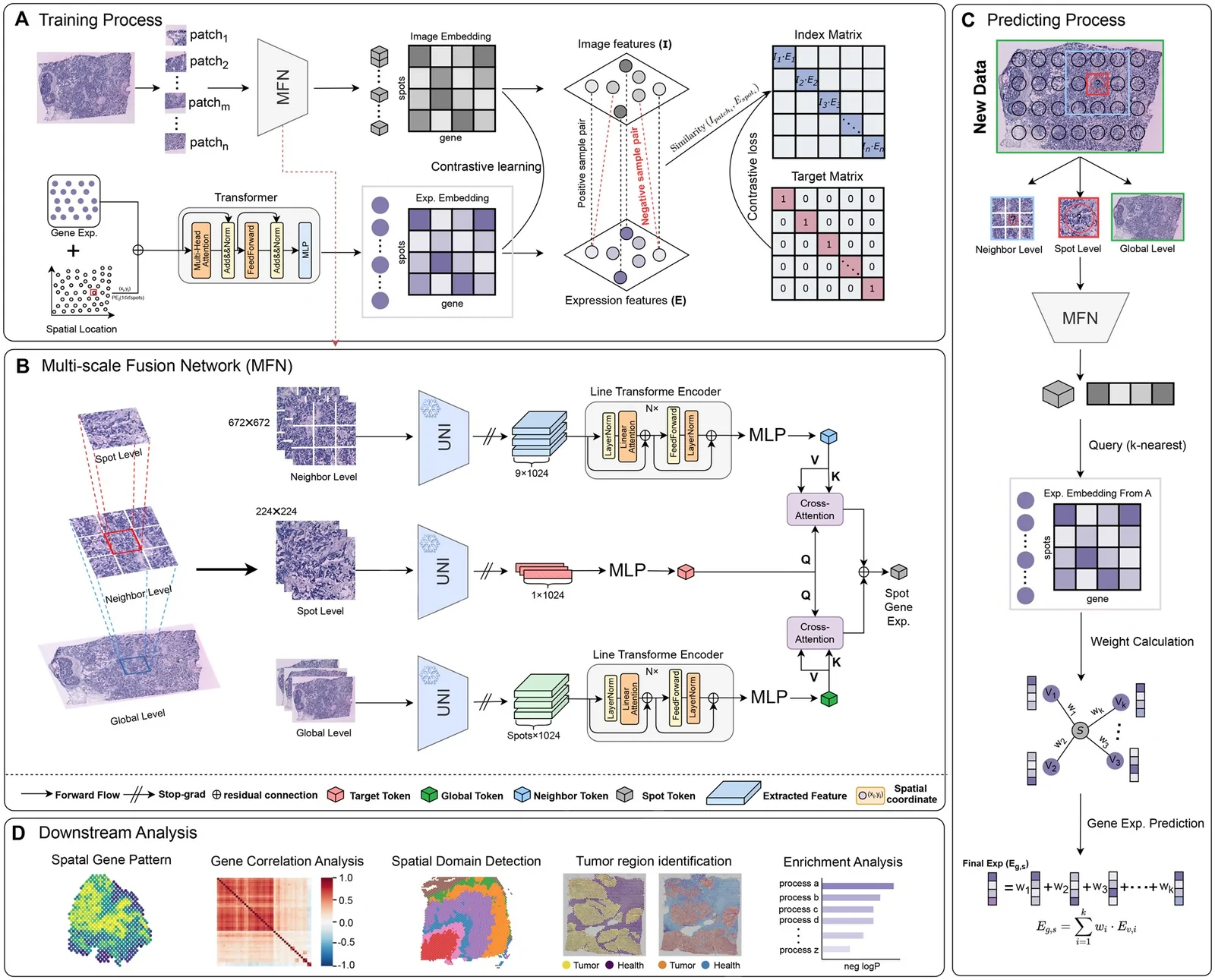
Overview of the HisCMCL Framework. (A) Training process: HisCMCL extracts image features via MFN and fuses gene expression with spatial data using Transformer self-attention. It then applies contrastive learning between image and expression features to maximize similarity for positive pairs and minimize it for negative pairs. (B) Multi-scale Fusion Network (MFN): For each image patch, HisCMCL extracts features at three different scales. The encoder module learns features, while the cross-attention module integrates features from different scales. (C) Prediction process: The test image is processed by MFN to extract features, which are then matched with the trained expression features. The nearest *k* spots are queried, and their expression values are weighted and aggregated to predict gene expression. (D) Downstream analysis.

As shown in Fig. 1, our framework employs an image encoder to extract multi-scale visual features from H&E images and fuses them via multi-head cross-attention. Gene expression data together with spatial coordinates are encoded to obtain spatial expression representations. Image and expression features are aligned through contrastive learning by maximizing the cosine similarity of matched pairs and minimizing that of mismatched pairs. During inference, we retrieve the top-k most similar spatial spots based on cosine similarity and aggregate their gene expression profiles to predict the spatial gene expression of the query image.

### 2.2 Training process of the proposed HisCMCL

During the training process, we use two separate encoders to extract image features and expression features, and optimize the model parameters through contrastive learning to maximize the similarity between positive sample pairs while minimizing the similarity between negative sample pairs (Fig. 1A).

#### 2.2.1 Image and spot encoders

We extract 224 × 224 pixel image patches from H&E images according to the spot position coordinates in the ST data. For each extracted image patch $\mathrm{Patc}h_{i}$, we use the multi-scale fusion network (MFN) shown in Fig. 1C to extract the corresponding gene expression vector. Then, we combine the features of all image patches into an image embedding matrix $I\in R^{N\times m}$.

In addition to H&E images, we also use the gene expression data and positional information of the spots. After preprocessing, the gene expression data for each spot is represented as an N×m matrix $G_{\;\text{exp}\;}$, while the position of each spot is recorded in an N×2 matrix representing the (x,y) coordinates. First, the x-coordinate is transformed into a one-hot encoding matrix $P_{x}$ of size N×n, where *n* is the maximum number of x-coordinates across all tissue sections. Then, a learnable linear layer $W_{x}$ maps $P_{x}$ to an N×m matrix $L_{x}$, which is consistent in dimension with the spot feature matrix $G_{\;\text{exp}\;}\in R^{N\times m}$. Similarly, the y-coordinate is processed in the same way, yielding the corresponding N×m encoding matrix $L_{y}$. Finally, the gene expression matrix $G_{\;\text{exp}\;}$, the x-coordinate matrix $L_{x}$, and the y-coordinate matrix $L_{y}$ are fused and processed using a multi-head self-attention mechanism, as follows:


(1) $$z_{{\text{spot}}_{i}}=\text{MHSA}(G_{\;\text{exp}\;}+L_{x}+L_{y}).$$

It is then mapped to the expression embedding matrix $E\in R^{N\times m}$ via a projection layer:


(2) $$E_{{\text{spot}}_{i}}=\text{MLP}(z_{{\text{spot}}_{i}}).$$

The self-attention mechanism in HisCMCL integrates gene expression features with spatial location information. This multimodal representation integrates core gene expression data with the specific spatial context of each tissue point.

#### 2.2.2 Contrastive learning based on images and expression

Since the partitioning of H&E image patches is based on the position coordinates of the spots, spots and image patches at the same location naturally form positive sample pairs, while spots and image patches at different locations form negative sample pairs.

We adopt a contrastive learning approach to optimize the fusion of image information and gene expression data. In each batch consisting of N pairs of (image patch, spot), we simultaneously train both the image encoder and the spot encoder with the goal of constructing a multimodal embedding space. Specifically, the image is mapped through the image latent space, while gene expression is mapped through the gene expression latent space. Our objective is to maximize the similarity between the image embedding and the gene expression embedding, especially for positive sample pairs (i.e., image patches and their corresponding spots), while minimizing the similarity between negative sample pairs (i.e., image patches and non-corresponding spots). Specifically, we first calculate the cosine similarity between the image embedding $h_{\text{patch}i}$ and the spot embedding $h_{{\text{spot}}_{i}}$, defined as:


(3) $$\text{cos}\;\_\text{sim}\left(I_{\mathrm{patc}h_{i}},E_{\mathrm{spo}t_{i}}\right)=\frac{I_{\mathrm{patc}h_{i}}\cdot E_{\mathrm{spo}t_{i}}^{T}}{\left|\left|I_{\mathrm{patc}h_{i}}\right|\right|\left|\left|E_{\mathrm{spo}t_{i}}\right|\right|}.$$

To distinguish between positive and negative pairs, we fine-tune the contrastive loss of CLIP (Radford *et al.* 2021) by introducing a target matrix (identity matrix), which identifies the correct (patch, spot) pairs in the training batch. The diagonal elements represent positive sample pairs (labeled as 1), and the non-diagonal elements represent negative sample pairs (labeled as 0). The target matrix target is defined as:


(4) $$\begin{array}{c} \mathrm{targe}t_{ij}=\{\begin{array}{cc} 1, & {\text{if}\;\text{patch}}_{i}{\;\text{and}\;\text{spot}}_{j}\;\text{form}\;a\;\text{positive}\;\text{pair}\\ 0, & {\text{if}\;\text{patch}}_{i}{\;\text{and}\;\text{spot}}_{j}\;\text{form}\;a\;\text{negative}\;\text{pair} \end{array}, \end{array}$$

Next, we define two loss components. Loss image is used to compute the similarity between image patches and spot embeddings, while Loss spot is used to compute the similarity between spot embeddings and image patch embeddings. The formulas are as follows:


(5) $${\text{Loss}}_{\mathrm{image}}=-\sum_{i=1}^{N}\sum_{j=1}^{N}{\text{target}}_{ij}\;\text{log}\;\left({\;\text{cos}\;}_{\text{sim}}\left(I_{\mathrm{patc}h_{i}},E_{\mathrm{spo}t_{i}}\right)\right)$$

(6) $${\text{Loss}}_{\mathrm{spot}}=-\sum_{i=1}^{N}\sum_{j=1}^{N}{\text{target}}_{ij}\;\text{log}\;\left({\;\text{cos}\;}_{\text{sim}}\left(E_{\mathrm{spo}t_{i}},I_{\mathrm{imag}e_{i}}\right)\right)$$

Finally, the overall loss is computed by taking the weighted average of these two losses:


(7) $${\text{Loss}}_{\mathrm{total}}=\lambda \times {\text{Loss}}_{\mathrm{image}}+\left(1-\lambda \right)\times {\text{Loss}}_{\mathrm{spot}},$$

Where λ is a hyperparameter that controls the relative weight of the image and spot losses. By minimizing this total loss, the model effectively adjusts its parameters to extract more accurate image and gene expression information, optimizing the distinction between positive and negative sample pairs.

### 2.3 Multi-scale fusion network (MFN)

#### 2.3.1 Multi-scale feature extraction

Due to the large size of WSIs, relying solely on the full-image information is insufficient to fully capture their morphological features. While WSIs provide rich global context, there is still a significant gap between the global view and the localized information at the patch level. To bridge this gap, we divide the WSI into multiple 224 × 224 pixel patches based on the locations of the spots, with each patch corresponding to a target spot. For each extracted image patch ${\text{Patch}}_{i}$, we select the k nearest patches around it and merge them into a neighbor representation.

As shown in Fig. 1B, our model extracts image patches from three hierarchical levels of WSIs and uses the pre-trained UNI (Chen *et al.* 2024) as the feature extractor. UNI (Chen *et al.* 2024) is a general-purpose foundation model pre-trained on a wide range of WSI datasets, specifically designed for pathology image analysis. For each patch, UNI extracts a feature vector $P_{i}$ of size 1×1024.


(8) $$P_{i}=\text{UNI}\left(\mathrm{patc}h_{i}\right),P_{i}\in R^{1\times 1024}.$$

At the spot level, we use the pre-trained UNI to embed the target spot into feature S, and then project it to the gene dimension using a projection layer. At the neighborhood level, we select the 8 nearest patches around the target spot, extract features from these 9 patches, and embed them into 9 feature vectors of size 1024. To enhance feature adaptability, we add a linear multi-head transformer after each UNI encoder to fuse these 9 feature vectors. Finally, the fused features are mapped to the gene expression dimension through a gene projection head, generating a context-enriched representation for the target spot *N*. At the global level, the feature extraction process is similar to that at the neighborhood level. In the global view, we analyze image patches of all spots in the WSI to provide a comprehensive representation of the tissue’s overall structure and spatial organization *G*.


(9) $$S_{i}=\text{MLP}\left(P_{i}\right),S_{i}\in R^{1\times m},$$

(10) $$N_{i}=\text{MLP}\left[\frac{1}{n}\sum_{j=1}^{n}\left(w_{j}P_{j}\right)\right],N_{i}\in R^{1\times m},$$

Where *m* is the number of highly variable genes for expression prediction.

#### 2.3.2 Linear transformer encoder

To integrate the multiple feature vectors extracted at the neighborhood and global levels, we adopt a linear Transformer (Parcollet *et al.* 2024) to address the high time complexity of traditional Transformers when calculating pairwise attention weights. Traditional Transformers have a time complexity of $O(T^{2})$, which is inefficient for processing long sequences. To improve efficiency, we reduce the complexity to O(T) by approximating self-attention. Given *m* input vectors $A=[a_{1},\ldots ,a_{n}]\in R^{n\times d}$, where d=1024, each input vector is first transformed into a high-dimensional representation $B=[b_{1},\ldots ,b_{n}]$ using a nonlinear function g(·). Then, all $b_{i}$ are aggregated into a global vector t, which is concatenated with each $a_{i}$, followed by a transformation with GELU activation to yield the final output.


(11) $$t=\frac{1}{n}\sum_{j=1}^{n}g_{1}\left(y_{j}\right),\;b_{j}=g_{3}\left(g_{2}\left(a_{j}\right)⊕t\right),$$

Where $g_{1}(\cdot )$, $g_{2}(\cdot )$, and $g_{3}(\cdot )$ are nonlinear transformation functions, and ⊕ represents the residual connection.

#### 2.3.3 Multi-level fusion with multi-head cross-attention

To enable efficient information fusion between features extracted at different scales, we use a multi-head cross-attention mechanism. Specifically, we design two parallel attention modules, both of which use the target location as the query (*Q*). One module uses the neighborhood location’s token as the key ($K_{n}$) and value ($V_{n}$), while the other uses the global location’s token as the key ($K_{g}$) and value ($V_{g}$). Subsequently, the outputs of the two cross-attention branches corresponding to the neighborhood scale and the global scale are initially fused through element-wise addition. Then, the fused feature is fed into a dedicated fusion module consisting of a linear projection layer, LayerNorm, ReLU activation function, and Dropout. After adjustment, it is projected back to the original feature dimension, and finally the final gene expression feature of the target position is obtained.


(12) $$\text{CrossAttention}\left(Q,K,V\right)=\text{Softmax}\left(\frac{QK^{T}}{\sqrt{d}}\right)V,$$

(13) $$f_{n}=\text{CrossAttention}\left(Q,K_{n},V_{n}\right),$$

(14) $$f_{g}=\text{CrossAttention}\left(Q,K_{g},V_{g}\right),$$

(15) $$f_{\text{final}}=\text{Merge}\left(f_{n},f_{g}\right)\in R^{1\times m},$$

Where $f_{n}$ and $f_{g}$ refer to the results of the neighborhood and global tokens relative to the target location. $f_{\text{final}}$ represents the final gene expression after fusion.

### 2.4 Predicting process of the proposed HisCMCL

As shown in Fig. 1C, given a new test H&E image, we divide it into N image patches of 224 × 224 pixels based on the positions of the spots. For the prediction process, we take one image patch as an example. First, we use the 224 × 224 pixel patch as the spot-level scale; then, we extract a 672 × 672 pixel neighborhood-level scale centered around this patch; finally, the entire H&E image size is used as the global-level scale. Through the MFN, we integrate the features from these three different scales into a feature vector $I_{\mathrm{patc}h_{i}}^{\mathrm{new}}$ and map it to the gene dimension.

Next, we calculate the cosine similarity between the test image feature $I_{\mathrm{patc}h_{i}}^{\mathrm{new}}$ and all spot features $E\in R^{N\times m}$, ensuring that the spot features remain consistent with those from the training process. Then, we retrieve the top K spot features that are most similar to the test image.

We calculate the Euclidean distance between the image patch feature and the k spot features retrieved from the query, thereby assigning a weight to each spot. The calculation formula is as follows:


(16) $$d(u,v)=\sqrt{\sum_{i=1}^{m}{\left(u_{i}-v_{i}\right)}^{2}},$$

Where u is the feature vector of the test image patch, v is the expression feature vector obtained during the training process, and *m* represents the dimensionality of the feature space. Finally, based on the weighted distances of the k queried spot gene expressions and similarity scores, the gene expression value of the test image patch $E_{g,s}$ is inferred. The weight calculation formula is as follows:


(17) $$w(u_{i},v)=\frac{d{\left(u_{i},v\right)}^{-2}}{\sum_{u_{j}\in N_{v}}d{\left(u_{j},v\right)}^{-2}}.$$

(18) $$E_{g,s}=\sum_{i=1}^{k}w_{i}\cdot E_{v,i},$$

Where $E_{g,s}$ represents the predicted gene expression value of the spot corresponding to the query patch, and $E_{v,i}$ represents the gene expression value of the reference sample.

## 3 Experimental results

### 3.1 Data resources and preprocessing

We evaluated our method on four publicly available multi-slice ST datasets (Table 1): two human breast cancer (BC) datasets with different resolutions, a cutaneous squamous cell carcinoma (cSCC) dataset (Ji *et al.* 2020), and a human dorsolateral prefrontal cortex (DLPFC) dataset (Maynard *et al.* 2021). Dataset details are in Section S2 of Supplementary Material, available as supplementary data at *Bioinformatics* online.

**Table 1:** Summary of all datasets used in this study.

Datasets	WSIs	Resolution	Spots	Tissue	Platform	References
Her2ST	32	100 µm	11538	breast	ST	(Andersson *et al.* 2021a)
cSCC	12	100 µm	8671	skin	ST	(Ji *et al.* 2020)
Alex	9	55 µm	24714	breast	10x	(Janesick *et al.* 2023)
DLPFC	12	55 µm	47631	brain	10x	(Maynard *et al.* 2021)

To reduce potential overfitting due to the limited dataset size, we use cross-validation across four ST datasets, ensuring no overlap of samples from the same patient between training and testing sets. Specifically, we adopt a leave-one-patient-out cross-validation method, where one patient’s sample is used for validation, and the remaining samples for training. This approach ensures a fair evaluation and consistency across all patients.

In gene selection and preprocessing, we identify highly variable genes shared across all slices in the training set, filter out low-expression genes, and normalize and log-transform the expression data. We then construct a stable and consistent gene feature set for each dataset for model training and evaluation. Detailed procedures are provided in Section S6 of Supplementary Material, available as supplementary data at *Bioinformatics* online.

### 3.2 Baseline methods

We compared the proposed HisCMCL model with eight advanced baselines, divided into two categories: (1) image feature-based methods, including STnet (He *et al.* 2020), Bleep (Xie *et al.* 2023), His2ST (Zeng *et al.* 2022), HisToGene (Pang *et al.* 2021), THItoGene (Jia *et al.* 2024), TRIPLEX (Chung *et al.* 2024) and STMCL (Shi *et al.* 2025c). (2) spatial topology-based method, STAGE (Li *et al.* 2024). The details of the baseline methods can be found in Section S3 of Supplementary Material, available as supplementary data at *Bioinformatics* online.

### 3.3 Evaluation metrics

Our evaluation metrics include pearson correlation coefficient (PCC), mean squared error (MSE), structural similarity index (SSIM), and adjusted rand index (ARI). We use two forms of PCC for comprehensive evaluation. PCC(M) is the average pearson correlation coefficient across all highly variable genes, reflecting the overall prediction performance. PCC(H) focuses on the top 50 most highly expressed highly variable genes to evaluate the prediction accuracy on key genes. Section S4 of Supplementary Material, available as supplementary data at *Bioinformatics* online provides detailed descriptions of all evaluation metrics.

### 3.4 Experiment settings

All experiments were conducted on an NVIDIA RTX 4090 GPU with the AdamW optimizer. Detailed experimental settings, including hyperparameters and configurations, are summarized in the supplementary materials (Table S4, available as supplementary data at *Bioinformatics* online). Implementation details are provided in Section S5 of Supplementary Material, available as supplementary data at *Bioinformatics* online.

### 3.5 HisCMCL improves the accuracy of spatial gene expression prediction

To evaluate the performance of HisCMCL, we analyzed multiple datasets, including the Her2ST breast cancer dataset (32 tissue slices), the cSCC dataset (12 slices), the Alex dataset (9 slices), and the DLPFC dataset (12 slices). We used leave-one-out cross-validation to assess the accuracy of gene expression prediction. Specifically, for each dataset, one slice was used as the test set, and the remaining slices were used for training. For each tissue slice, we calculated the average PCC (PCC(M)) for all genes, the average PCC (PCC(H)) for the top 50 highly expressed genes, and the MSE for PCC(M). We compared HisCMCL with eight state-of-the-art baselines for spatial gene expression prediction. Given that gene expression prediction tasks emphasize capturing relative changes, we preferred evaluation metrics related to PCC. As shown in Table 2, HisCMCL achieved the highest PCC(M) and PCC(H) across all four datasets. Specifically, HisCMCL’s PCC(M) was 16.01%, 7.14%, 49.2%, and 11.47% higher than the second-ranked STMCL method, and PCC(H) was 14.68%, 11.09%, 46.43%, and 14.41% higher, respectively. Additionally, HisCMCL’s MSE was the lowest across all datasets.

**Table 2:** For the Her2ST, cSCC, Alex, and DLPFC datasets, the average Pearson correlation coefficient PCC(M) for all genes, as well as the average Pearson correlation coefficient PCC(H) and mean squared error (MSE) for the top 50 genes, were calculated and compared with the baselines of eight different models. Bold: best results.

		Her2ST			cSCC			Alex			DLPFC		
Type	Method	PCC(M)	PCC(H)	MSE	PCC(M)	PCC(H)	MSE	PCC(M)	PCC(H)	MSE	PCC(M)	PCC(H)	MSE
Expression	STAGE	0.2048 ±0.018	0.3174 ±0.016	0.5961 ±0.007	0.2753 ±0.016	0.3494 ±0.014	0.4982 ±0.010	0.1009 ±0.015	0.1937 ±0.012	0.2574 ±0.007	0.2908 ±0.019	0.4157 ±0.016	0.4821 ±0.011
Image (Local)	STNet	0.0144 ±0.022	0.0563 ±0.017	0.5312 ±0.011	0.0012 ±0.021	0.0018 ±0.022	0.6806 ±0.005	0.0009 ±0.016	0.0452 ±0.013	0.4721 ±0.009	0.0418 ±0.017	0.0556 ±0.014	0.5269 ±0.012
	BLEEP	0.1873 ±0.015	0.2909 ±0.016	0.6015 ±0.016	0.2449 ±0.023	0.3122 ±0.027	0.5163 ±0.007	0.0912 ±0.014	0.1756 ±0.011	0.2593 ±0.009	0.2871 ±0.015	0.4102 ±0.014	0.4871 ±0.007
	His2ST	0.1441 ±0.011	0.1852 ±0.016	0.5135 ±0.009	0.1838 ±0.017	0.2175 ±0.021	0.6748 ±0.017	0.0958 ±0.013	0.1569 ±0.017	0.3788 ±0.008	0.1916 ±0.015	0.2021 ±0.016	0.5163 ±0.014
	STMCL	0.2142 ±0.007	0.3513 ±0.017	0.5897 ±0.014	0.3137 ±0.021	0.4146 ±0.024	0.4402 ±0.009	0.1054 ±0.013	0.2074 ±0.020	0.2529 ±0.006	0.2928 ±0.019	0.4205 ±0.015	0.4795 ±0.009
Image (Global)	HistoGene	0.0723 ±0.026	0.0832 ±0.020	0.5202 ±0.013	0.0771 ±0.028	0.0919 ±0.018	0.6805 ±0.014	0.0315 ±0.015	0.0984 ±0.014	0.4565 ±0.011	0.0529 ±0.017	0.0706 ±0.021	0.5221 ±0.012
	THItoGene	0.1725 ±0.021	0.2809 ±0.019	0.5012 ±0.011	0.2373 ±0.009	0.2719 ±0.012	0.6546 ±0.007	0.0873 ±0.009	0.1370 ±0.012	0.3672 ±0.010	0.2221 ±0.018	0.2578 ±0.016	0.4976 ±0.009
Multi-scale	TRIPLEX	0.2147 ±0.014	0.3497 ±0.017	0.5228 ±0.007	0.3074 ±0.018	0.4092 ±0.026	0.3268 ±0.009	0.1097 ±0.012	0.2473 ±0.014	0.3318 ±0.011	0.2063 ±0.017	0.3528 ±0.011	0.4202 ±0.012
	Ours	**0.2379** **±0.012**	**0.4029** **±0.019**	**0.4035** **±0.008**	**0.3361** **±0.018**	**0.4606** **±0.017**	**0.2716** **±0.009**	**0.1573** **±0.011**	**0.3037** **±0.016**	**0.2434** **±0.010**	**0.3264** **±0.014**	**0.4811** **±0.017**	**0.3798** **±0.011**

To further verify the spatial structural consistency of predicted expression patterns, we calculated gene-wise SSIM between predicted and ground-truth 2D expression maps using the top 50 highly variable genes. HisCMCL achieves superior SSIM performance across all datasets, demonstrating its strong capability in preserving the spatial structure and continuity of gene expression. Detailed results are provided in Section S9 of Supplementary Material, available as supplementary data at *Bioinformatics* online.

### 3.6 HisCMCL provides accurate predictions of marker genes

**Figure 2:**
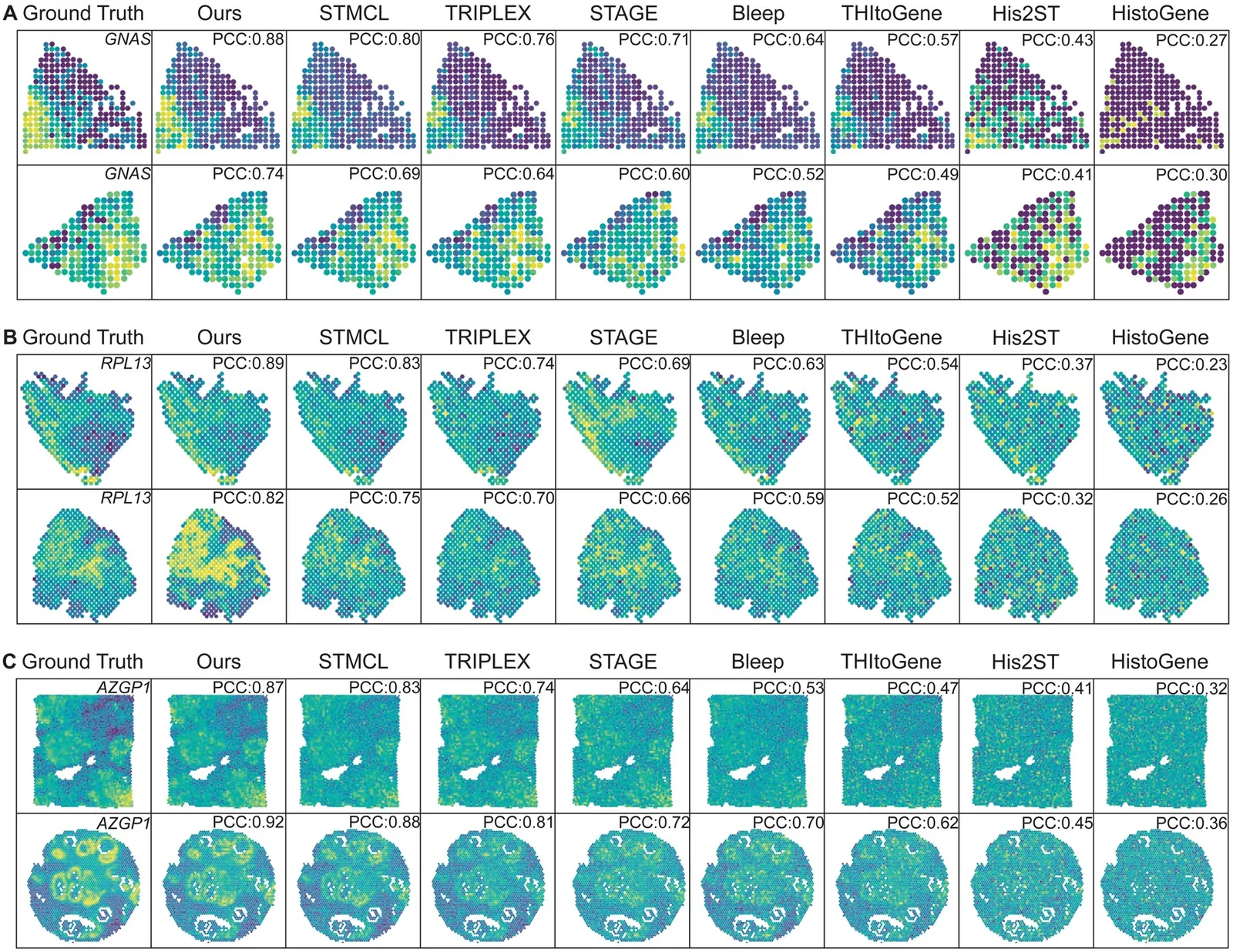
HisCMCL excels in generating tissue gene expression profiles. (A) Expression of the marker gene *GNAS* on the B1 and C1 slices from the Her2ST dataset. (B) Expression of the marker gene *RPL13* on the P10_ST_rep3 and P2_ST_rep2 slices from the cSCC dataset. (C) Expression of the marker gene *AZGP1* on the block2 and FFPE slices from the Alex dataset. Each panel shows the ground truth and predictions from representative methods. All three genes are known markers.

**Figure 3:**
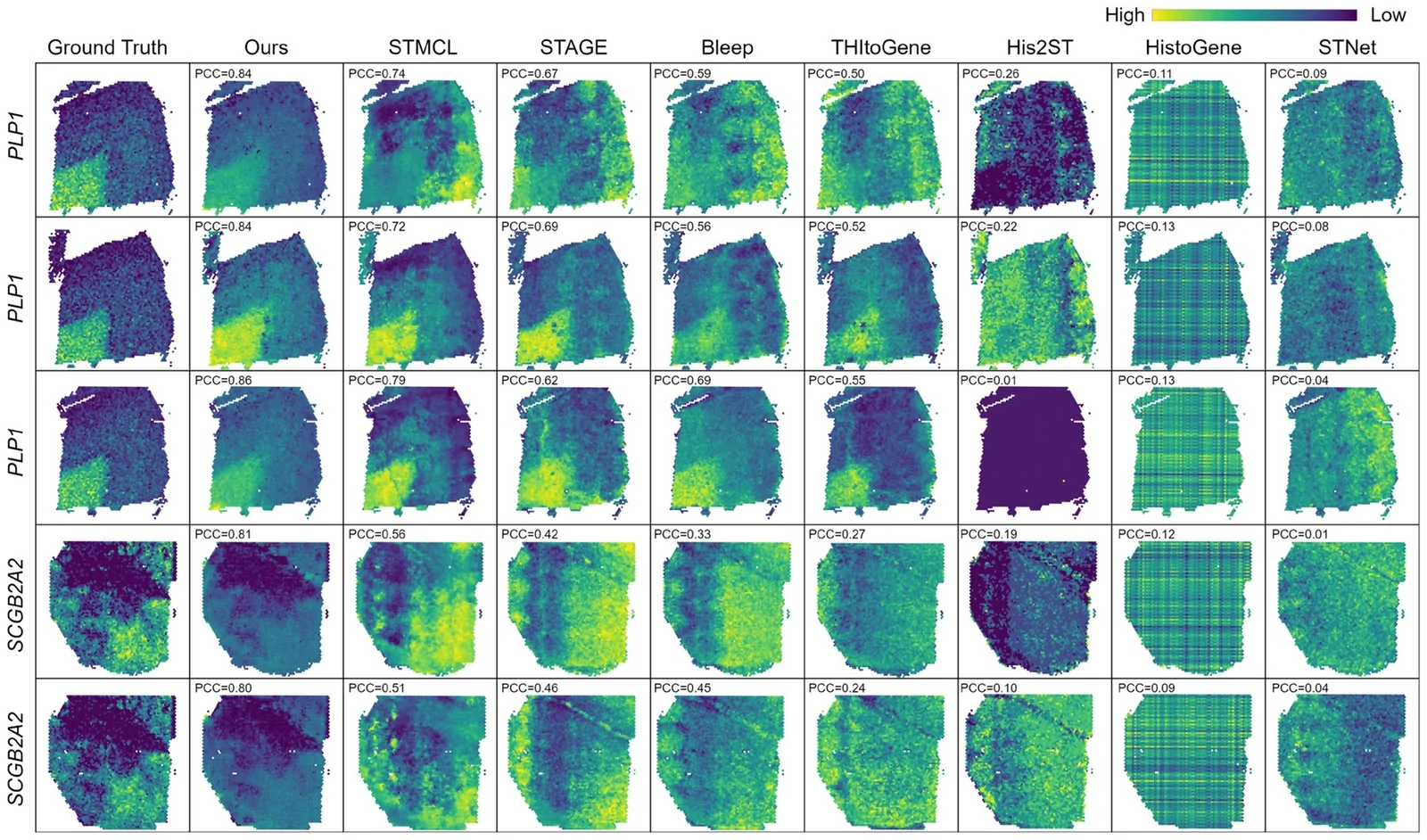
Visualization of predicted expression patterns for marker genes on the DLPFC dataset. Results are shown for HisCMCL and seven baseline methods across five different tissue sections.

To provide an intuitive evaluation of gene expression prediction across various baseline methods, we selected several marker genes closely associated with diseases from four datasets for visualization analysis. Additionally, we presented the Pearson correlation coefficient (PCC) values between predicted and true gene expressions. The results clearly demonstrate that HisCMCL significantly outperforms other models. Using the Her2ST and DLPFC datasets as examples, we visualized the gene *GNAS* (Jin *et al.* 2019) in the Her2ST dataset and the genes *PLP1* (Campagnoni and Skoff 2001) and *SCGB2A2* (Zafrakas *et al.* 2006) in the DLPFC dataset. In the Her2ST dataset, the PCC value for the marker gene *GNAS* (Jin *et al.* 2019) predicted by HisCMCL was 0.88, which is 10% higher than the second-ranking method, STMCL (see Fig. 2A). In the DLPFC dataset, we selected 5 different slices for visualization. The gene *PLP1* (Campagnoni and Skoff 2001) was visualized on slices 151674–151676, while *SCGB2A2* (Zafrakas *et al.* 2006) was visualized on slices 151507 and 151508, as shown in Fig. 3. HisCMCL significantly outperformed the other seven baseline methods in terms of PCC across these five slices. In addition to these quantitative results, a qualitative inspection of the color distribution in the images revealed that HisCMCL better replicated the underlying gene expression patterns, highlighting its exceptional predictive ability.

### 3.7 HisCMCL better preserves gene co-expression patterns

Considering that noise may obscure true gene co-expression relationships, we conducted gene co-expression analysis to assess whether HisCMCL can capture complex gene-gene interactions rather than just matching expression distributions. This is crucial for identifying functionally related genes and regulatory networks. We performed the analysis on test slice data from four datasets. Specifically, we calculated the mean expression value of each gene across all spots within each slice and selected the top 50 highly expressed genes for further analysis. Selecting highly expressed genes helps ensure robust correlation analysis and focuses on genes that may have functional significance. Next, we computed the pcc between each pair of selected genes, resulting in a 50 × 50 correlation matrix. The analysis was conducted on both observed gene expression and predictions from different methods to compare their performance. Finally, the gene-gene correlation matrices for the four tissue slices were visualized using heatmaps, with pcc values represented by a color spectrum ranging from dark blue to dark red.

**Figure 4:**
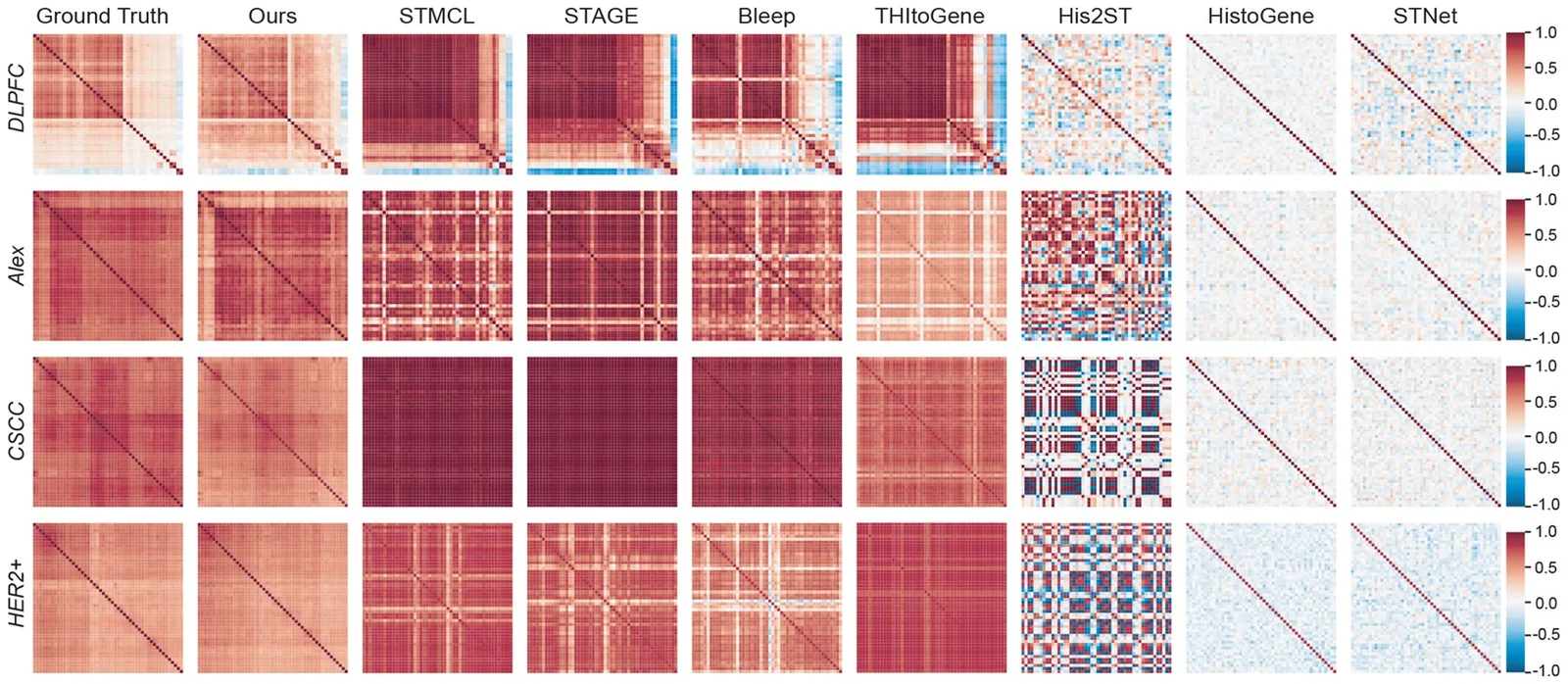
Gene co-expression analysis across tissue sections using different methods. The heatmap shows the PCC values between the top 50 highly expressed genes, ranging from −1 (dark blue) to 1 (dark red). This analysis compares the correlation patterns between observed gene expression and predictions from eight methods across four datasets.

As shown in Fig. 4, the observed gene expression reveals distinct block structures, indicating gene co-expression relationships. Our correlation analysis demonstrates that HisCMCL outperforms other methods in three key aspects:

- Accurately preserving the main correlation blocks, including highly correlated regions as well as uncorrelated or negatively correlated regions.
- Appropriately enhancing the visibility of co-expression patterns.
- Sensitively detecting correlation strength, capturing subtle variations in gene relationships.

In contrast, other methods, especially His2ST (Zeng *et al.* 2022), HistoGene (Pang *et al.* 2021), and STNet (He *et al.* 2020), show limited ability to capture correlation blocks and tend to oversimplify relationship patterns. HisToGene performs the worst, displaying scattered association patterns and introducing noisy correlations that are not present in the observed data.

### 3.8 HisCMCL reconstructs spatial domains from predicted gene expression

**Figure 5:**
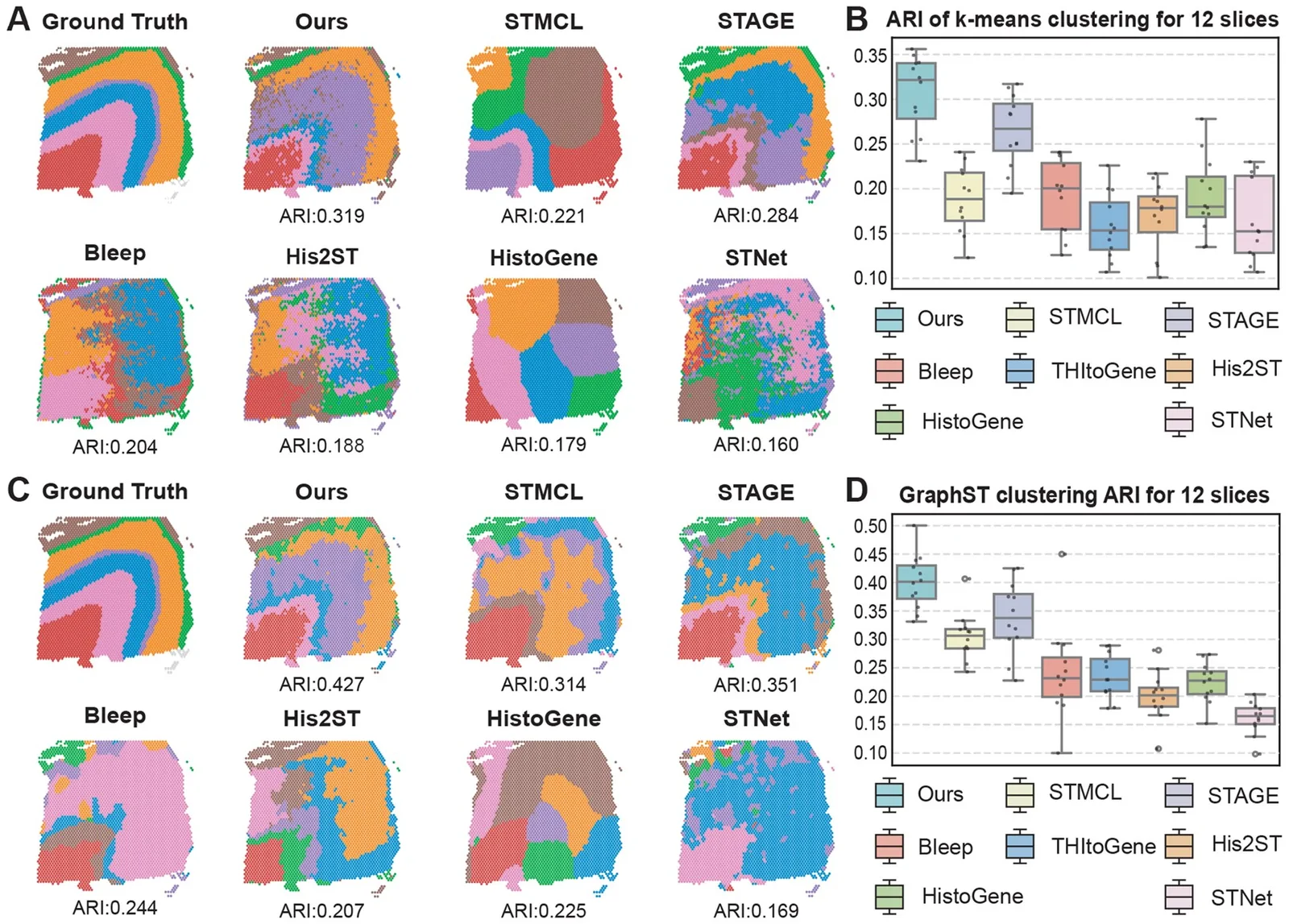
The accuracy of spatial domain identification from both raw data and data generated by HisCMCL, STMCL, STAGE, Bleep, His2ST, HisToGene, and ST-Net was compared on the DLPFC dataset. (A) shows the spatial domain visualization on slice 151,673 using the K-means clustering method, with HisCMCL and other methods. (B) presents a performance comparison of recognition accuracy between HisCMCL and other methods using the K-means method. (C) demonstrates the spatial domain identification visualization on slice 151,673 when using the GraphST clustering method for HisCMCL and other methods. (D) compares the recognition accuracy of HisCMCL and other methods using the GraphST method.

To evaluate the performance of HisCMCL in spatial domain recognition tasks, we compared 12 tissue slices from the DLPFC dataset, which were annotated for spatial transcriptomics by pathologists. We applied K-means and GraphST (Long *et al.* 2023) clustering methods to both the original and generated data, using pathologist annotations as the reference, with ARI as the evaluation metric. Fig. 5A and C show the clustering results of K-means and GraphST (Long *et al.* 2023) methods on the 151,673 slice of the DLPFC dataset. The results indicate that our method’s clustering is closer to the manual annotations, and the ARI values are significantly higher than those of other methods. Fig. 5B and D display the ARI box plots for K-means and GraphST (Long *et al.* 2023) clustering methods across the 12 slices. It is evident that HisCMCL outperforms the second-best method, STAGE, in both the highest and average ARI values. These results demonstrate that HisCMCL achieves greater accuracy and robustness in spatial domain recognition, effectively capturing the spatial structure of the data.

### 3.9 HisCMCL can accurately identify tumor regions

**Figure 6:**
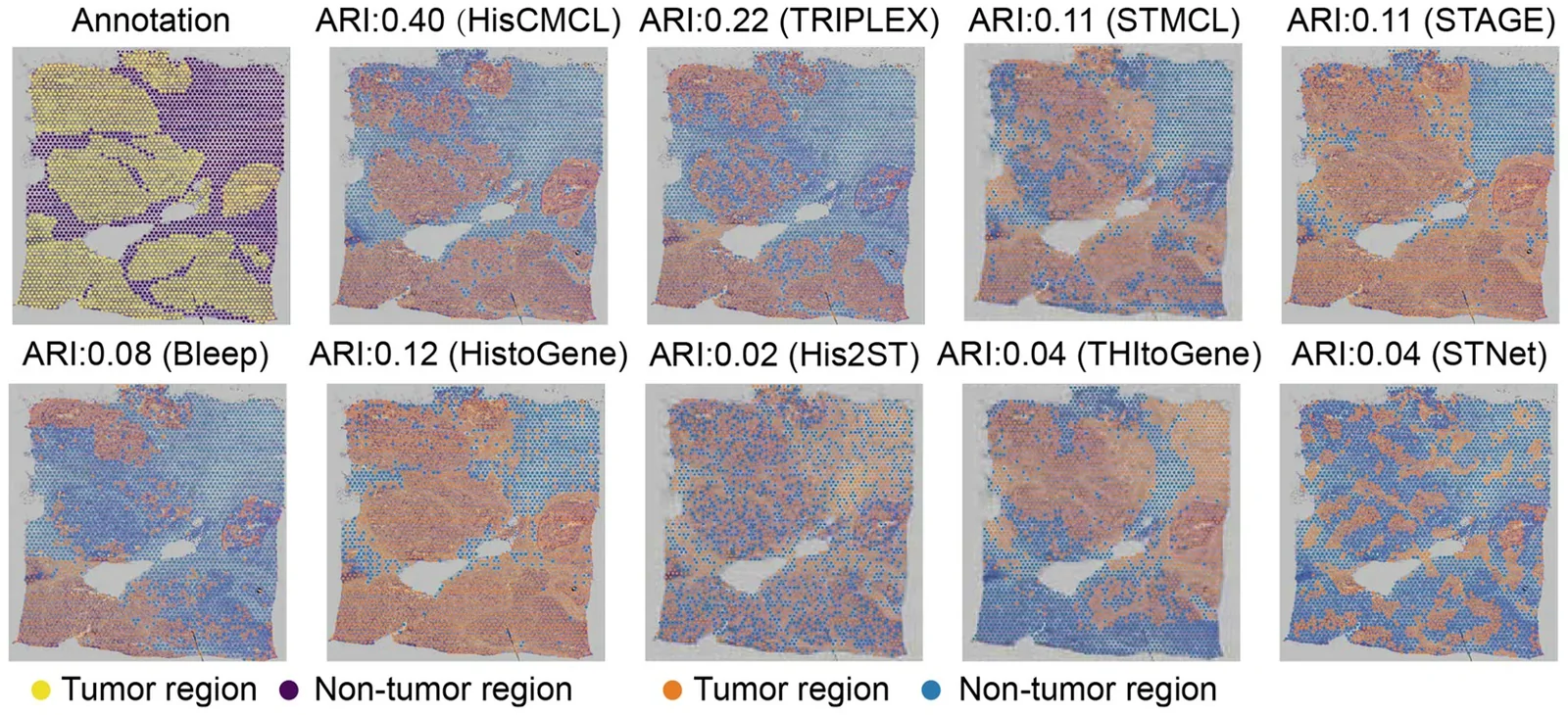
Clustering analysis of the predicted gene expression profiles in the Alex dataset was performed. Based on the tissue regions annotated by pathologists, the spots were classified into cancerous and normal tissue regions.

The gene expression profile of spots in ST data reflects the functional state and pathological heterogeneity of tissues, serving as a crucial foundation for accurately identifying tumor regions. To evaluate the tumor region recognition capability of different methods, we performed clustering on the predicted gene expression in the Alex dataset (Janesick *et al.* 2023). Using the tissue regions annotated by pathologists as the gold standard, we classified the spots into tumor and non-tumor areas. The results, shown in the Fig. 6, demonstrate that HisCMCL achieved the highest ARI, significantly outperforming other methods, showcasing its excellent accuracy in tumor region recognition.

### 3.10 HisCMCL can explore the biological functions of breast cancer data

**Figure 7:**
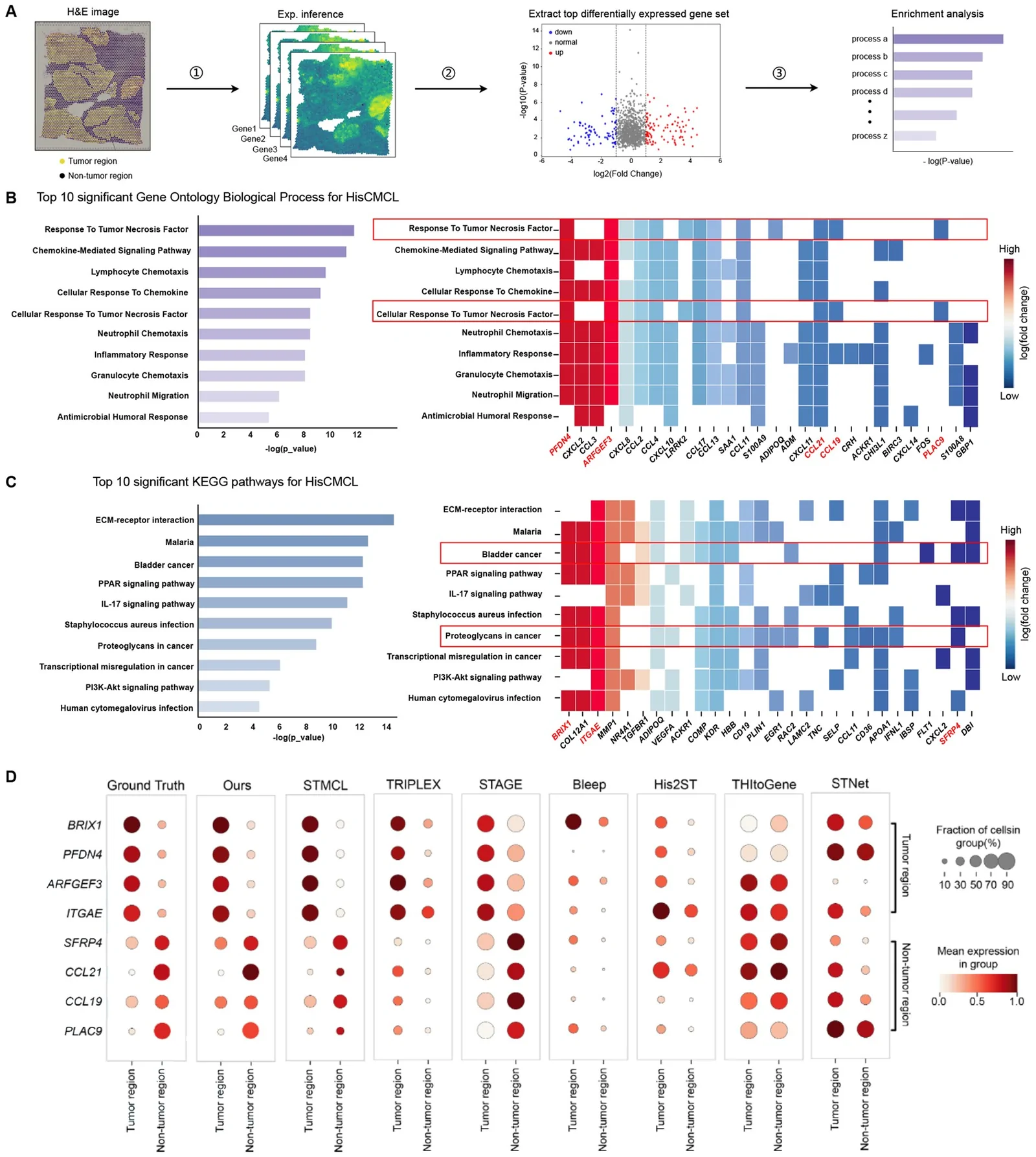
Based on the gene expression predicted by HisCMCL on the Alex dataset block1 slice, GO: BP and KEGG pathway enrichment analyses were performed for differentially expressed genes in tumor regions. (A) shows the workflow for identifying differentially expressed genes and performing enrichment analysis based on HisCMCL-predicted gene expression. (B) displays the *P* value of the top 10 significant GO: BP terms identified by HisCMCL in tumor regions, along with the corresponding GO: BP pathway enrichment analysis heatmap. The impact of related differential genes on these pathways is analyzed by calculating the log(fold change), and the pathways highlighted in red are our main focus. (C) shows the *P* value of the top 10 significant KEGG pathways identified by HisCMCL in tumor regions, along with the corresponding KEGG pathway enrichment analysis heatmap. (D) displays the bubble plot of 8 highly expressed differential genes in tumor and non-tumor regions using different prediction methods.

To evaluate the biological relevance of gene expression predicted by HisCMCL, we performed differential expression analysis on the block1 slice of the Alex dataset based on the predicted expression levels, identifying genes with significant differences between tumor and non-tumor regions. The Wilcoxon rank-sum test in scanpy was used for differential expression testing; genes with an adjusted *P* value < 0.0001 and $\left|{\;\text{log}\;}_{2}\left(\text{FC}\right)\right|>1$ were defined as significantly differentially expressed genes. We further identified upregulated (${\;\text{log}\;}_{2}\left(\text{FC}\right)>1$) and downregulated (${\;\text{log}\;}_{2}\left(\text{FC}\right)<-1$) gene sets for subsequent enrichment analysis (Fig. 7A).

We performed GO: BP enrichment analysis on the differentially expressed genes in the tumor region, identifying the top 10 biological processes with the smallest adjusted *P* value (Fig. 7B). Several processes are related to cancer progression, such as response to tumor necrosis factor (Laha *et al.* 2021). We used the Sepal tool (Andersson *et al.*, 2021b) to rank differentially expressed genes in these processes and selected the top 28 genes for heatmap analysis. High expression of *PFDN4* and *ARFGEF3* was found in these pathways, which may be linked to tumor cell proliferation and microenvironment remodeling. In contrast, *CCL21*, *CCL19*, and *PLAC9* were downregulated in these pathways, suggesting potential immune evasion in the tumor microenvironment (Hauser and Legler 2016, Wang *et al.* 2021).

KEGG enrichment analysis revealed the top 10 significant pathways, with bladder cancer and proteoglycans in cancer closely associated with cancer (Fig. 7C). The heatmap analysis showed high expression of *BRIX1* and *ITGAE* in the tumor region, indicating tumor cell proliferation and active tumor-infiltrating lymphocytes (TILs), respectively. *SFRP4*, a Wnt signaling antagonist, was downregulated, suggesting a loss of tumor-suppressive function (Bhuvanalakshmi *et al.* 2018).

To further analyze gene expression, we visualized it using a bubble plot (Fig. 7D). HisCMCL accurately identified high-expression genes in cancer regions, while other methods like STNet misclassified some non-cancer genes. In addition, we performed comparative analyses of HisCMCL with STMCL and TRIPLEX based on GO: BP and KEGG enrichment results, which are presented in the supplementary materials (Fig. S3, available as supplementary data at *Bioinformatics* online). These analyses reveal that the two competing methods are less capable of identifying key cancer-associated pathways and show lower enrichment significance than HisCMCL. These results highlight the potential of HisCMCL in providing biological insights into tumor mechanisms and its value as a predictive framework in cancer research.

## 4 Ablation study

To validate the effectiveness of our model design, we conducted ablation experiments on key components including image encoder, multi-scale branches, positional encoding, distance metric, and Top-*k* fusion hyperparameter. The contribution of each architectural module was validated through ablation experiments, the results of which are available in Section S8 of Supplementary Material, available as supplementary data at *Bioinformatics* online.

We first evaluated different image encoders, including ResNet50, DenseNet121, TITAN (Ding *et al.* 2025), CONCH (Lu *et al.* 2024), and UNI. The results (Table S5) show that the Transformer-based pathology model UNI achieves the highest PCC and lowest MSE on all datasets owing to its domain-specific pre-training.

We then verified the multi-scale branches and found that fusing all three scales yields the best performance; removing the Neighbor branch significantly reduces accuracy, highlighting the importance of local spatial interactions (Table S6).

In addition, positional encoding helps the model capture spatial dependencies in transcriptomic data and improves performance across all metrics (Table S7).

Finally, we analyzed the hyperparameter *k* in the Top-*k* similar spots weighted fusion strategy, confirming that k=200 achieves stable and optimal performance on all datasets, which verifies its rationality (Fig. S4, available as supplementary data at *Bioinformatics* online).

## 5 Conclusion

In this study, we propose HisCMCL, a multimodal deep learning framework that combines local and global features with contrastive learning to improve the accuracy of spatial gene expression prediction. HisCMCL enhances prediction performance by using a multi-level information fusion strategy to extract rich contextual features from histological images and incorporating spatial information through positional encoding. The model optimizes the cosine similarity of positive sample pairs using contrastive learning, bringing correctly matched image patches and spot samples closer, while minimizing the cosine similarity of negative sample pairs to push mismatched samples apart, thereby effectively integrating image data. Experimental results demonstrate that HisCMCL significantly outperforms existing methods across multiple datasets, showcasing its ability to interpret cancer-specific overexpressed genes and identify key spatial regions annotated by pathologists.

Although the proposed method achieves promising performance, there is still room for further improvement. The Top-K retrieval and weighted fusion strategy adopted in the inference phase fails to fully account for the high heterogeneity of the tumor microenvironment. Under circumstances where visual features are similar but gene expression profiles differ, the robustness of the model can be further enhanced. In the future, we will optimize the retrieval and fusion process by integrating adaptive weighting and attention mechanisms to mitigate the adverse effects caused by inappropriate matches. We will also explore end-to-end prediction architectures to improve the stability and applicability of the model in complex tissue scenarios.

In summary, HisCMCL provides an effective technical approach for spatial gene expression prediction, which remedies the limitations of conventional methods to a certain extent and offers a referential direction for relevant future research.

## Supplementary Material

btag342_Supplementary_Data

## Data Availability

The source code for this study is publicly available at GitHub (https://github.com/wenwenmin/HisCMCL) as well as on Zenodo with DOI (https://doi.org/10.5281/zenodo.19735383). All datasets used in this study can be accessed and downloaded from Zenodo (https://zenodo.org/records/18030446).
