# Supplementary material for: HisCMCL: cross-modal contrastive learning with hierarchical multi-scale fusion for spatial expression prediction
Source: Bioinformatics. 2026 May 29;42(6):btag342. doi: 10.1093/bioinformatics/btag342 (PMC13596053; doi:10.1093/bioinformatics/btag342)
Supplement: Supplementary file 1 [file btag342_supplementary_data.pdf]

Supplementary Material for

HisCMCL: Cross-Modal Contrastive Learning with Hierarchical Multi-Scale Fusion for Spatial Expression Prediction

S1 Code Availability and Reproducibility

- **Code Availability via GitHub Link.** The complete code, datasets, and pre-trained models, along with detailed instructions, are available at: https://github.com/wenwenmin/HisCMCL. A comprehensive README file in the GitHub repository provides guidance on how to use the model to test the data and obtain results.
- **Implementation Details.** More implementation and technical details can be found in Supplementary Section S4, including the specific architecture of the model, a detailed description of the training process, and the rationale behind the selection of hyperparameters.
- **Data Availability.** Due to GitHub's storage limitations, only a portion of the data is available via the GitHub link. All datasets used in this study have been uploaded to the Zenodo database(https://zenodo.org/records/18030446).

S2 Dataset Description

We selected four publicly available spatial transcriptomics (ST) datasets, which cover various tissue types, disease contexts, and experimental platforms, as summarized in Table S1.

Her2ST (ST, Breast Cancer): The Her2ST dataset (Andersson et al., 2020) includes spatial transcriptomics data from HER2-positive invasive ductal carcinoma (IDC) samples from seven patients. Each slice was obtained from fresh-frozen breast tissue using the standard ST protocol, with a spot diameter of 100 μm and an inter-spot distance of 200 μm. The dataset contains 32 samples, covering the tumor core and surrounding regions, providing high-quality histological images and corresponding spot-level gene expression matrices for each sample.

cSSC (ST, Cutaneous Squamous Cell Carcinoma): The cSSC dataset (Ji et al., 2020) consists of formalin-fixed paraffin-embedded (FFPE) cutaneous squamous cell carcinoma samples from four pa-

tients, analyzed using spatial transcriptomics technology (with a center-to-center spacing of 100 μm and a spot diameter of 150 μm). This dataset includes 12 samples, and the tissue slices exhibit a complex tumor microenvironment, including keratinized tumor nests, stromal regions, and immune cell infiltrates.

Alex (10x Visium, Breast Cancer): The Alex dataset (Janesick et al., 2023) includes 9 breast cancer tissue slices obtained from 4 patients in Swarbrick's laboratory. These samples were analyzed using spatial transcriptomics technology, revealing the gene expression distribution within breast cancer tissues. Each sample contains multiple spatial spots, measured using standard spatial transcriptomics techniques, providing valuable data for studying the molecular characteristics and spatial structure of breast cancer.

DLPFC (10x Visium, Dorsolateral Prefrontal Cortex): The DLPFC dataset (Maynard et al., 2021) includes 12 tissue sections from the dorsolateral prefrontal cortex (DLPFC) of three individuals. Each sample contains 74,631 spots, with each spot representing a 55 μm area. The dataset reveals the complex regional architecture of the DLPFC, including different cortical layers, neuronal populations, glial cells, and vascular structures. Gene expression data for 33,536 genes are provided for each spot, offering valuable resources for studying gene expression distribution and spatial organization in different regions of the brain.

Table S1: Spatial transcriptomics datasets aggregated by individual. Each individual entry reports the individual information across multiple slices, along with the corresponding platform information.

Dataset	Platform	Individuals	Tissue	Condition	Samplex	Spot Diameter (μm)	Preservation	Reference
Her2ST	ST	Patient A	Breast	Cancer	5	100	Fresh Frozen	(Andersson et al., 2020)
	ST	Patient B	Breast	Cancer	6	100	Fresh Frozen	same as above
	ST	Patient C	Breast	Cancer	6	100	Fresh Frozen	same as above
	ST	Patient D	Breast	Cancer	6	100	Fresh Frozen	same as above
	ST	Patient E	Breast	Cancer	3	100	Fresh Frozen	same as above
	ST	Patient F	Breast	Cancer	3	100	Fresh Frozen	same as above
	ST	Patient G	Breast	Cancer	3	100	Fresh Frozen	same as above
cSCC	ST	Patient 1	Skin	Cancer	3	100	FFPE	(Ji et al., 2020)
	ST	Patient 2	Skin	Cancer	3	100	FFPE	same as above
	ST	Patient 3	Skin	Cancer	3	100	FFPE	same as above
	ST	Patient 4	Skin	Cancer	3	100	FFPE	same as above
Alex	10x Visium	Patient 1	Breast	Cancer	2	55	Fresh Frozen	(Janesick et al., 2023)
	10x Visium	Patient 2	Breast	Cancer	2	55	Fresh Frozen	same as above
	10x Visium	Patient 3	Breast	Cancer	4	55	Fresh Frozen	same as above
	10x Visium	Patient 4	Breast	Cancer	1	55	FFPE	same as above
DLPFC	10x Visium	Individual 1	Brain	Normal	4	55	Fresh Frozen	(Maynard et al., 2021)
	10x Visium	Individual 2	Brain	Normal	4	55	Fresh Frozen	same as above
	10x Visium	Individual 3	Brain	Normal	4	55	Fresh Frozen	same as above

S3 Baseline Methods

In this study, we conducted a comprehensive comparison between our proposed HisCMCL framework and eight state-of-the-art gene expression prediction methods: STNet (He et al., 2020), HisToGene (Pang et al., 2021), His2ST (Zeng et al., 2022), THItoGene (Jia et al., 2024), BLEEP (Xie et al., 2023), STAGE (Li et al., 2024), TRIPLEX (Chung et al., 2024), and STMCL (Shi et al., 2025). These methods represent the current advances in the field of spatial transcriptomics, each adopting different strategies to address the complex task of predicting gene expression from histological images and spatial transcriptomics data.

STNet (He et al., 2020) combines spatial transcriptomics with histology images and uses a deep convolutional network to directly predict gene expression from H&E-stained images. The method employs a DenseNet-121 backbone pretrained on ImageNet, with a fully connected regression head for expression prediction. Each 224×224 patch centered on a spatial spot is used as input, and the model learns to map visual information from the image to the corresponding expression profile. In our implementation, we preserve the original network configuration and normalization strategy to ensure results consistent with the published framework.

HisToGene (Pang et al., 2021) introduces a vision Transformer–based approach for predicting spatial gene expression from histology images. The method divides the tissue image into patches that are fed into a Vision Transformer module to capture global contextual information through self-attention. The Transformer-encoded features are then regressed to gene expression values, enabling the model to learn a mapping from image content to expression profiles. Our implementation follows the original Transformer architecture and feature extraction pipeline.

His2ST (Zeng et al., 2022) further enhances spatial neighborhood modeling on top of HisToGene. This method extracts local image texture features with convolutional modules and uses Transformer and graph neural network components to capture global context and adjacency interactions between spots. By integrating these diverse visual and spatial representations, the model predicts the expression levels at each location more comprehensively. Our reproduction adheres to the multi-module design and training strategy of the original work.

THItoGene (Jia et al., 2024) proposes a hybrid neural network architecture to learn spatial expression patterns from histology images. It combines dynamic convolution and attention mechanisms on top of a Transformer backbone, incorporating graph attention networks to strengthen spatial relationship modeling. The input consists of spot-centered image patches and positional encodings, and the output is the predicted gene expression vector. Our implementation maintains the original module design and feature fusion strategy.

BLEEP (Xie et al., 2023) develops a bi-modal embedding framework that aligns image and expression features into a shared low-dimensional space via contrastive learning. The method uses ResNet-50 as the image encoder and an MLP for expression encoding, and trains them with a contrastive loss to bring modalities into alignment. During inference, expression values are predicted through nearest-neighbor lookup in the joint embedding space based on image embeddings. Our implementation adopts the dual-encoder structure and contrastive alignment scheme from the original work.

STAGE (Li et al., 2024) jointly models spatial coordinates and gene expression as a supervised latent generative process by embedding spatial locations and expression patterns into a continuous latent space. The model learns to predict expression profiles from spatial information through a generator module, enforcing spatial smoothness and consistency in the predictions. Our implementation follows the spatial embedding strategy and generative loss formulation described in the original design.

TRIPLEX (Chung et al., 2024) proposes a multi-resolution deep learning framework for expression prediction. It extracts features at three levels—target spot patch, neighboring context, and global tissue view—via separate encoders, then fuses these multi-scale representations to predict expression values. This design allows the model to capture both local details and broader spatial context simultaneously, improving the quality of the predicted expression maps. Our reproduction retains the multi-path encoding and fusion process of the original model.

STMCL (Shi et al., 2025) introduces a multimodal contrastive learning method that integrates histology images, spot expression data, and spatial coordinates into a unified representation space. The model optimizes consistency between modalities using contrastive loss and incorporates positional encodings to jointly learn representations from image and expression information. At inference, the combined embeddings are used to output gene expression predictions, enabling the model to capture synergistic relationships across image, spatial, and expression modalities. Our implementation adheres to the original contrastive objectives and embedding design.

To quantitatively assess the performance differences among the methods, we conducted Wilcoxon signed-rank tests on the PCC correlation scores between HisCMCL and each of the other comparison methods across all datasets (see Table S2). All p-values in the table are less than 0.001, indicating that the performance differences between HisCMCL and the compared methods are highly significant in a statistical sense, which demonstrates that HisCMCL's prediction performance is significantly superior to that of the other baseline methods on these datasets.

Table S2: Wilcoxon signed-rank test p-values comparing HisCMCL with competing methods across different datasets. Each p-value is computed based on paired comparisons of PCC scores across all tissue slices within the dataset.

P-value	Her2ST	cSCC	Alex	DLPFC
HisCMCL vs. STNet	<0.001	<0.001	<0.001	<0.001
HisCMCL vs. HisToGene	<0.001	<0.001	<0.001	<0.001
HisCMCL vs. His2ST	<0.001	<0.001	<0.001	<0.001
HisCMCL vs. THItoGene	<0.001	<0.001	<0.001	<0.001
HisCMCL vs. Bleep	<0.001	<0.001	<0.001	<0.001
HisCMCL vs. STAGE	<0.001	<0.001	<0.001	<0.001
HisCMCL vs. TRIPLEX	<0.001	<0.001	<0.001	<0.001
HisCMCL vs. STMCL	<0.001	<0.001	<0.001	<0.001

Table S3: HistoGene and STMCL are retrained using the same UNI embeddings across all four datasets.

Method	Her2ST			cSCC			Alex			DLPFC		
	PCC(M)	PCC(H)	MSE	PCC(M)	PCC(H)	MSE	PCC(M)	PCC(H)	MSE	PCC(M)	PCC(H)	MSE
HistoGene-UNI	0.1156 ±0.053	0.1703 ±0.037	0.4815 ±0.014	0.1196 ±0.032	0.2194 ±0.021	0.5063 ±0.016	0.0719 ±0.028	0.1286 ±0.010	0.4079 ±0.016	0.1028 ±0.019	0.2295 ±0.017	0.4975 ±0.014
STMCL-UNI	0.2197 ±0.014	0.3864 ±0.026	0.4311 ±0.013	0.3025 ±0.027	0.4361 ±0.022	0.3525 ±0.007	0.1256 ±0.013	0.2755 ±0.018	0.2517 ±0.012	0.3027 ±0.015	0.4374 ±0.018	0.4417 ±0.010
Ours	**0.2379 ±0.012**	**0.4029 ±0.019**	**0.4035 ±0.008**	**0.3361 ±0.018**	**0.4606 ±0.017**	**0.2716 ±0.009**	**0.1573 ±0.011**	**0.3037 ±0.016**	**0.2434 ±0.010**	**0.3264 ±0.014**	**0.4811 ±0.017**	**0.3798 ±0.011**

S4 Evaluation metrics

To comprehensively evaluate the prediction performance of the HisCMCL model and other baseline methods on four datasets, we conduct data analysis by comparing the original data with the predicted data, and adopt metrics such as Pearson Correlation Coefficient (PCC), Mean Squared Error (MSE), and Adjusted Rand Index (ARI) for performance evaluation.

PCC is a statistical measure used to assess the strength of the linear relationship between two variables, commonly used to evaluate the correlation between predicted and true values. We calculate the average PCC for all genes (PCC(M)) and the average PCC for highly predicted genes (PCC(H)). Highly predicted genes are identified through a cross-validation ranking process, where the top 50 genes are determined based on their average ranking across all folds. The calculation method for PCC is as follows:

$$\mathrm{PCC} = \frac{\mathrm{Cov}\left(X_{real}, X_{pred}\right)}{\sqrt{\mathrm{Var}\left(X_{real}\right) \times \mathrm{Var}\left(X_{pred}\right)}}, \tag{1}$$

where $\mathrm{Cov}(\cdot)$ represents covariance, $\mathrm{Var}(\cdot)$ represents variance, and X_{real} and X_{pred} are the observed and predicted gene expression, respectively.

MSE measures the average squared difference between predicted and true values. The smaller the MSE, the better the model performance. The formula for MSE is:

$$\mathrm{MSE} = \frac{1}{N} \sum_{i=1}^{N} \left(X_{\mathrm{real},i} - X_{\mathrm{pred},i}\right)^2, \tag{2}$$

where $X_{real,i}$ and $X_{pred,i}$ are the observed and predicted expression values of the i_{th} gene, respectively.

ARI is used to evaluate the clustering performance, measuring the consistency between the clustering results and the true labels. A higher value indicates better consistency. Its formula is:

$$\mathrm{ARI} = \frac{\sum_{ij} \binom{n_{ij}}{2} - \mathbb{E}[\sum_{ij} \binom{n_{ij}}{2}]}{\frac{1}{2}\left[\sum_i \binom{a_i}{2} + \sum_j \binom{b_j}{2}\right] - \mathbb{E}[\sum_{ij} \binom{n_{ij}}{2}]}, \tag{3}$$

where n_{ij} denotes the number of samples belonging to both the true class i and the predicted class j, and a_i and b_j denote the total number of samples in the true class i and predicted class j, respectively, $\mathbb{E}[\cdot]$ represents the expected value to calculate the expected number of such sample pairs under random cluster assignments.

S5 Additional Implementation Details

In this study, all experiments were conducted on an NVIDIA RTX 4090 GPU, using the PyTorch 2.5.1 and Python 3.10.15 environment. Our model was trained using the Adam optimizer. The batch size was set based on requirements, and gradient accumulation was applied to avoid out-of-memory issues. During

Table S4: Hardware/software environment and training hyperparameters.

Hardware/Software Configurations and Hyperparameters		
Category	Item / Library	Version / Specification
Hardware	CPU	Intel Xeon w5-2455x
	GPU	1 $\times$ NVIDIA RTX 4090 (24 GB)
	RAM	64 GB
	Operating System	Windows 11
Software	Python	3.10.15
	CUDA	12.1
	PyTorch	2.5.1
Libraries	NumPy	1.26.4
	SciPy	1.13.0
	scikit-learn	1.5.2
	pandas	2.2.2
	matplotlib	3.9.0
	scanpy	1.9.8
	anndata	0.10.8
Hyperparameters	batch_size	128
	learning rate	1×10^{-3}
	heads_num	32
	heads_dim	256
	image_embedding_dim	1024
	projection_dim	256
	temperature	1.0

training, each batch of data undergoes a forward pass, computes the loss, and performs backpropagation. To save GPU memory, the model regularly clears the cache.

To ensure the reproducibility and transparency of the experiments, we have systematically organized and fully documented all key implementation details, including training hyperparameters, optimizer settings, learning rate schedules, software and hardware configurations, and network architectures. These detailed settings are summarized in a unified table for readers to consult and reproduce the entire experimental pipeline of this study.

The model implementation is based on several different neural network architectures, using the Transformer model to extract both image and spatial information. Specifically, multiple image encoders, including ResNet, DenseNet, VIT, and UNI, were used to extract features at different scales. To further process spatial information, multi-head attention mechanisms and feed-forward networks were employed, enabling the effective fusion of image and spatial features.

Additionally, the model adopted a multi-scale feature extraction strategy to handle images with different resolutions. During training, the model adjusts based on different image scales, utilizing attention mechanisms to weight and fuse features from different scales, thus enhancing performance across various resolutions.

After training, the model saves its optimal parameters and configurations to ensure the training process can be replicated. All training details, hyperparameter configurations, and model states are saved and a unique saving path is generated based on the dataset and model configurations to ensure the reproducibility of the experiments.The detailed experimental environment is shown in Table S4.

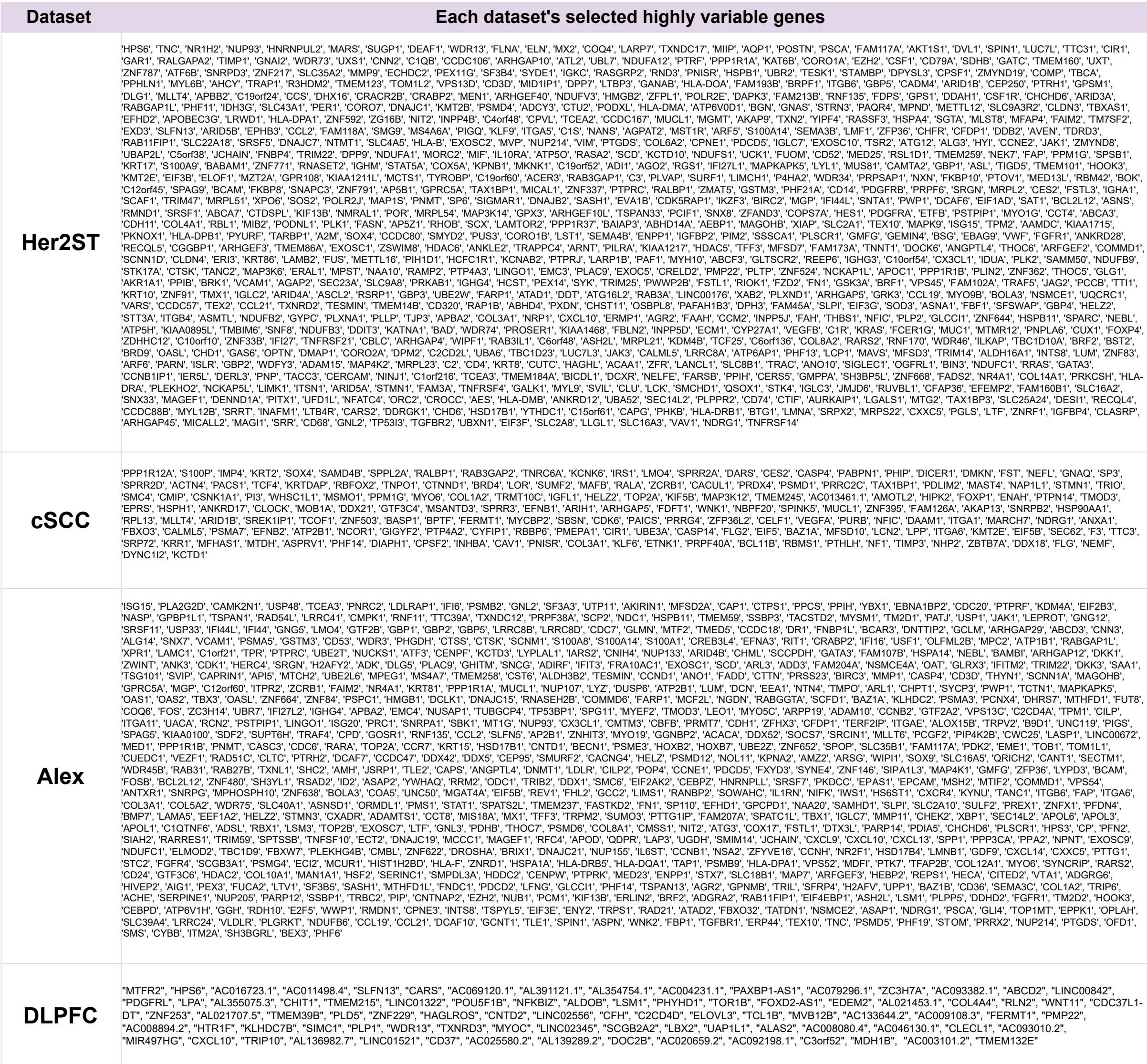

Fig. S1: Selected genes used across different datasets.

S6 Genes Selection

To select genes for model training, we first compiled a list of genes shared across all spatial transcriptomics slices in the training set. For each individual slice, we then identified the top 1,000 genes with the highest variability and removed genes that were expressed in fewer than 1,000 spots across the entire dataset. For each spot's expression profile, gene counts were normalized by dividing the UMI count of each gene by the total UMI count of the spot and multiplying by 1,000,000, followed by natural log transformation of the normalized values. After this preprocessing, 785, 171, 685, and 84 highly variable genes were

retained as the final feature set for the Her2ST, cSCC, Alex, and DLPFC datasets, respectively. The summary statistics of the selected gene sets are presented in Fig. S1.

S7 Enrichment analysis

To assess the biological relevance of gene expression predicted by HisCMCL, we performed differential expression analysis on the Alex dataset to identify genes that significantly differed between the regions labeled as "invasive cancer" and "non-tumor tissue." These genes were then subjected to Gene Ontology Biological Process (GO:BP) pathway enrichment analysis and Kyoto Encyclopedia of Genes and Genomes (KEGG) pathway enrichment analysis.

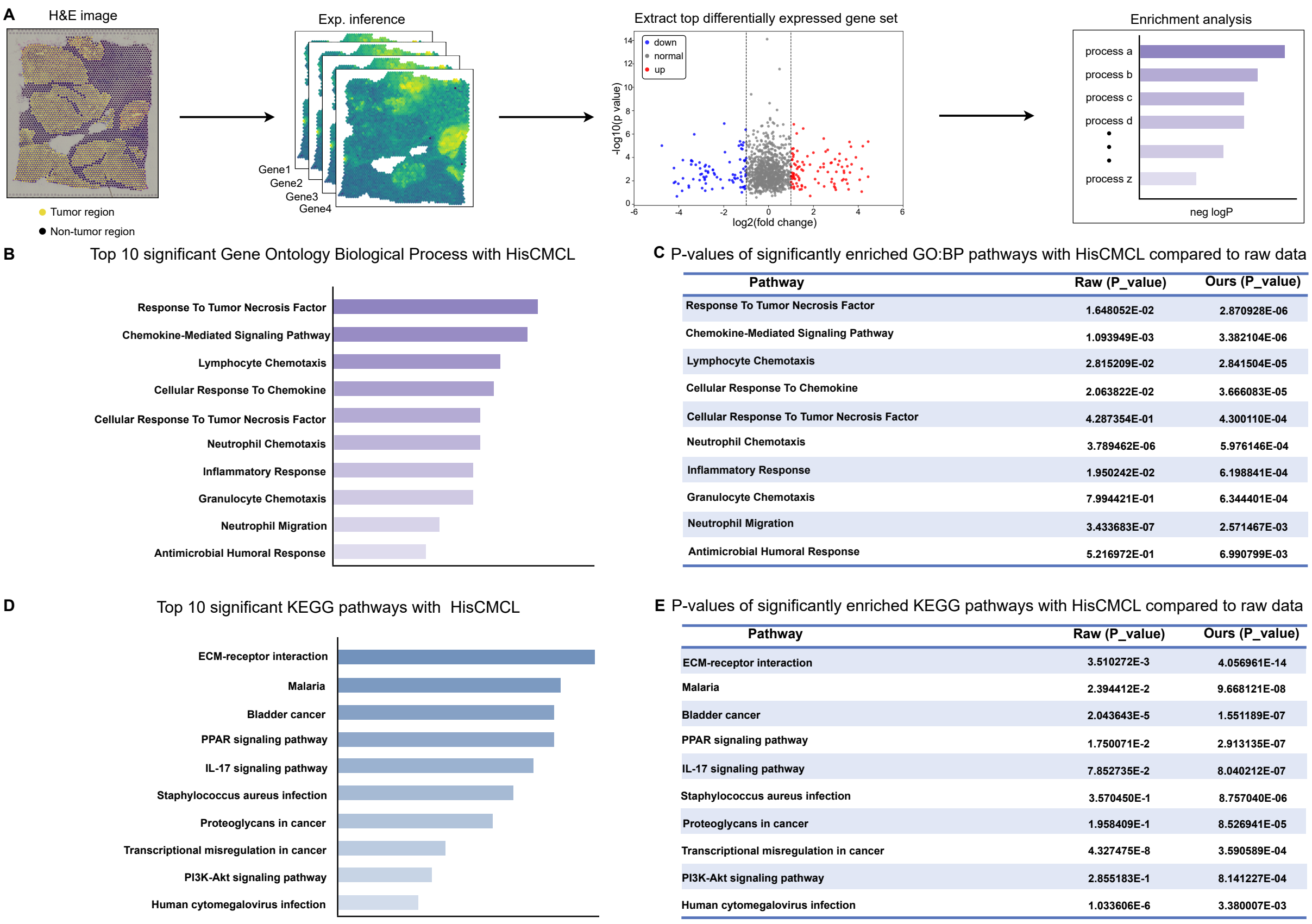

C P-values of significantly enriched GO:BP pathways with HisCMCL compared to raw data

Pathway	Raw (P_value)	Ours (P_value)
Response To Tumor Necrosis Factor	1.648052E-02	2.870928E-06
Chemokine-Mediated Signaling Pathway	1.093949E-03	3.382104E-06
Lymphocyte Chemotaxis	2.815209E-02	2.841504E-05
Cellular Response To Chemokine	2.063822E-02	3.666083E-05
Cellular Response To Tumor Necrosis Factor	4.287354E-01	4.300110E-04
Neutrophil Chemotaxis	3.789462E-06	5.976146E-04
Inflammatory Response	1.950242E-02	6.198841E-04
Granulocyte Chemotaxis	7.994421E-01	6.344401E-04
Neutrophil Migration	3.433683E-07	2.571467E-03
Antimicrobial Humoral Response	5.216972E-01	6.990799E-03

E P-values of significantly enriched KEGG pathways with HisCMCL compared to raw data

Pathway	Raw (P_value)	Ours (P_value)
ECM-receptor interaction	3.510272E-3	4.056961E-14
Malaria	2.394412E-2	9.668121E-08
Bladder cancer	2.043643E-5	1.551189E-07
PPAR signaling pathway	1.750071E-2	2.913135E-07
IL-17 signaling pathway	7.852735E-2	8.040212E-07
Staphylococcus aureus infection	3.570450E-1	8.757040E-06
Proteoglycans in cancer	1.958409E-1	8.526941E-05
Transcriptional misregulation in cancer	4.327475E-8	3.590589E-04
PI3K-Akt signaling pathway	2.855183E-1	8.141227E-04
Human cytomegalovirus infection	1.033606E-6	3.380007E-03

Fig. S2: Based on gene expression predicted by HisCMCL on the Alex dataset, differential expression analysis of genes in tumor regions compared to other tissues. (**A**) A workflow diagram for identifying differentially expressed genes and performing enrichment analysis based on gene expression predictions by HisCMCL. (**B**) and (**C**) Show the P-values of significantly enriched GO:BP pathways identified by HisCMCL, compared to the raw dataset. (**D**) and (**E**) Show the P-values of significantly enriched KEGG pathways identified by HisCMCL, compared to the raw data.

As shown in Fig. S2, several canonical pathways closely associated with tumor initiation and progression were identified, such as such as the response to tumor necrosis factor (Laha et al., 2021) and the cellular response to tumor necrosis factor (López-Urrutia et al., 2017). Compared to the results based on the original expression data, the enrichment results derived from the predicted expression exhibited greater statistical significance, suggesting that the gene expression predicted by HisCMCL is more closely

aligned with the biological characteristics of cancerous tissue.

To further demonstrate the superiority of HisCMCL in identifying biologically meaningful pathways, we compared its enrichment performance with two state-of-the-art approaches, STMCL and TRIPLEX. As displayed in Fig. S3, although STMCL and TRIPLEX could capture a subset of tumor-related pathways, they yielded far fewer significantly enriched terms than HisCMCL. Importantly, several critical cancer-associated pathways, including bladder cancer, proteoglycans in cancer, and transcriptional misregulation in cancer, were not significantly enriched by these two methods. For the pathways that were successfully detected, such as ECM-receptor interaction and the PI3K-Akt signaling pathway, their corresponding P-values were generally higher than those from HisCMCL and the original data, implying weaker statistical confidence. These observations reveal that the expression profiles predicted by STMCL and TRIPLEX may deviate from genuine biological patterns, thus limiting their ability to reliably and comprehensively characterize tumor-related biological processes. In comparison, HisCMCL not only detected a larger number of significantly enriched GO:BP and KEGG pathways but also achieved stronger statistical significance across all key pathways, further verifying that its predictions are highly consistent and reliable with the biological features of cancer tissues.

A GO:BP pathway enrichment significance results across different methods

Pathway	Raw (P_value)	Ours (P_value)	STMCL (P_value)	TRIPLEX (P_value)
Response To Tumor Necrosis Factor	1.648052E-02	2.870928E-06	9.905786E-03	———
Chemokine-Mediated Signaling Pathway	1.093949E-03	3.382104E-06	4.379336E-04	1.488496E-04
Lymphocyte Chemotaxis	2.815209E-02	2.841504E-05	5.076577E-02	2.010664E-02
Cellular Response To Chemokine	2.063822E-02	3.666083E-05	2.104389E-03	3.096405E-02
Cellular Response To Tumor Necrosis Factor	4.287354E-01	4.300110E-04	4.260469E-02	———
Neutrophil Chemotaxis	3.789462E-06	5.976146E-04	5.976146E-04	2.565153E-03
Inflammatory Response	1.950242E-02	6.198841E-04	1.187158E-02	2.432069E-03
Granulocyte Chemotaxis	7.994421E-01	6.344401E-04	6.338107E-02	4.506931E-02
Neutrophil Migration	3.433683E-07	2.571467E-03	3.707827E-01	7.339147E-04
Antimicrobial Humoral Response	5.216972E-01	6.990799E-03	———	———

B KEGG pathway enrichment significance results across different methods

Pathway	Raw (P_value)	Ours (P_value)	STMCL (P_value)	TRIPLEX (P_value)
ECM-receptor interaction	3.510272E-03	4.056961E-14	3.700902E-04	4.794912E-03
Malaria	2.394412E-02	9.668121E-08	———	———
Bladder cancer	2.043643E-05	1.551189E-07	———	———
PPAR signaling pathway	1.750071E-02	2.913135E-07	3.197200E-04	1.882988E-04
IL-17 signaling pathway	7.852735E-02	8.040212E-07	5.512994E-03	4.260805E-02
Staphylococcus aureus infection	3.570450E-01	8.757040E-06	2.458324E-04	7.833112E-02
Proteoglycans in cancer	1.958409E-01	8.526941E-05	———	———
Transcriptional misregulation in cancer	4.327475E-08	3.590589E-04	1.557839E-03	———
PI3K-Akt signaling pathway	2.855183E-01	8.141227E-04	4.334794E-02	8.708672E-02
Human cytomegalovirus infection	1.033606E-06	3.380007E-03	1.090395E-04	6.483617E-04

Fig. S3: (A) shows the P-values of significantly enriched GO:BP pathways identified by HisCMCL compared with the results of raw data, STMCL and TRIPLEX. (B) shows the P-values of significantly enriched KEGG pathways identified by HisCMCL compared with the results of the aforementioned methods.

S8 Ablation study

Image Encoder. We compared UNI (Chen et al., 2024), TITAN (Ding et al., 2025), CONCH (Lu et al., 2024), DenseNet121, and ResNet (He et al., 2016) as image encoders, with the results shown in Table S5. UNI is a vision backbone model pre-trained specifically for histopathological images, and it performs the best overall, achieving the highest PCC across all datasets. The excellence of UNI is attributed to its Transformer architecture and domain-specific pre-training, enabling it to more accurately capture spatial patterns in ST data.

Table S5: Ablation studies of image encoders on the Her2ST, cSCC, Alex and DLPFC datasets.

Image Encoder	Her2ST			cSCC			Alex			DLPFC		
	PCC(M)	PCC(H)	MSE	PCC(M)	PCC(H)	MSE	PCC(M)	PCC(H)	MSE	PCC(M)	PCC(H)	MSE
Resnet50	0.2064 ±0.012	0.3146 ±0.013	0.6058 ±0.006	0.2994 ±0.007	0.4139 ±0.005	0.4385 ±0.004	0.0915 ±0.015	0.1906 ±0.014	0.2394 ±0.008	0.2789 ±0.014	0.4002 ±0.011	0.5269 ±0.004
DenseNet121	0.2152 ±0.020	0.3265 ±0.016	0.5821 ±0.004	0.3235 ±0.010	0.4249 ±0.009	0.4302 ±0.006	0.1058 ±0.011	0.2069 ±0.016	0.2373 ±0.006	0.2915 ±0.015	0.4123 ±0.010	0.4871 ±0.007
TITAN	0.2278 ±0.017	0.3486 ±0.023	0.5417 ±0.011	0.3049 ±0.021	0.4338 ±0.028	0.4158 ±0.010	0.1239 ±0.013	0.2357 ±0.027	0.2461 ±0.012	0.2894 ±0.032	0.4309 ±0.027	0.4328 ±0.016
CONCH	0.2306 ±0.008	0.3791 ±0.018	0.4642 ±0.028	0.3291 ±0.013	0.4421 ±0.019	0.3259 ±0.019	0.1477 ±0.012	0.2816 ±0.024	**0.2253 ±0.016**	0.3025 ±0.019	0.4618 ±0.020	0.3940 ±0.014
UNI	**0.2379 ±0.012**	**0.4029 ±0.019**	**0.4035 ±0.008**	**0.3361 ±0.018**	**0.4606 ±0.017**	**0.2716 ±0.009**	**0.1573 ±0.011**	**0.3037 ±0.016**	0.2434 ±0.010	**0.3264 ±0.014**	**0.4811 ±0.017**	**0.3798 ±0.011**

Multi-scale Branches. We evaluated whether the integration of information from Spot, Neighbor, and Global layers could improve the accuracy of gene expression prediction. As shown in Table S6, integrating all three layers of information achieved the best performance. Notably, excluding the Neighbor layer resulted in a significant decrease in PCC and a substantial increase in MSE, highlighting the critical impact of neighboring interactions on HisCMCL gene expression prediction.

Table S6: Ablation study of image feature fusion at different branch scales across all datasets.

Multiscale Features	Her2ST			cSCC			Alex			DLPFC		
	PCC(M)	PCC(H)	MSE	PCC(M)	PCC(H)	MSE	PCC(M)	PCC(H)	MSE	PCC(M)	PCC(H)	MSE
w/ Spot	0.2151 ±0.008	0.3789 ±0.013	0.6014 ±0.011	0.3177 ±0.021	0.4162 ±0.013	0.4418 ±0.011	0.1355 ±0.007	0.2464 ±0.015	0.2602 ±0.010	0.2869 ±0.010	0.4196 ±0.008	0.4262 ±0.004
w/ Spot+Neighbor	0.2211 ±0.011	0.3805 ±0.014	0.5974 ±0.012	0.3248 ±0.017	0.4275 ±0.014	0.4521 ±0.007	0.1479 ±0.009	0.2810 ±0.013	0.2569 ±0.009	0.3072 ±0.009	0.4437 ±0.014	0.39561 ±0.007
Ours	**0.2379 ±0.012**	**0.4029 ±0.019**	**0.4035 ±0.008**	**0.3361 ±0.018**	**0.4606 ±0.017**	**0.2716 ±0.009**	**0.1573 ±0.011**	**0.3037 ±0.016**	**0.2434 ±0.010**	**0.3264 ±0.014**	**0.4811 ±0.017**	**0.3798 ±0.011**

Positional Encoding. As shown in Table S7, the inclusion of positional encoding allows the transformer to recognize the position of each element in the sequence, helping the model capture sequential relationships and significantly improving overall performance.

Table S7: The impact of integrating positional encoding information into gene expression features on model performance.

Positional Encoding	Her2ST			cSCC			Alex			DLPFC		
	PCC(M)	PCC(H)	MSE	PCC(M)	PCC(H)	MSE	PCC(M)	PCC(H)	MSE	PCC(M)	PCC(H)	MSE
w/o PE	0.2159 ±0.008	0.3738 ±0.012	0.5736 ±0.010	0.3164 ±0.012	0.4469 ±0.008	0.3268 ±0.013	0.1463 ±0.009	0.2831 ±0.013	0.2724 ±0.007	0.3048 ±0.010	0.4627 ±0.011	0.4166 ±0.009
PE	**0.2379 ±0.012**	**0.4029 ±0.019**	**0.4035 ±0.008**	**0.3361 ±0.018**	**0.4606 ±0.017**	**0.2716 ±0.009**	**0.1573 ±0.011**	**0.3037 ±0.016**	**0.2434 ±0.010**	**0.3264 ±0.014**	**0.4811 ±0.017**	**0.3798 ±0.011**

Table S8: Ablation study of distance metrics in contrastive learning.

Distance	Her2ST			cSCC			Alex			DLPFC		
	PCC(M)	PCC(H)	MSE	PCC(M)	PCC(H)	MSE	PCC(M)	PCC(H)	MSE	PCC(M)	PCC(H)	MSE
L1	0.2098 ±0.014	0.3214 ±0.016	0.6009 ±0.009	0.3087 ±0.013	0.3901 ±0.009	0.4317 ±0.007	0.0911 ±0.011	0.1887 ±0.014	0.6162 ±0.014	0.2736 ±0.014	0.3945 ±0.016	0.5075 ±0.008
L2	0.2107 ±0.009	0.3325 ±0.012	0.5963 ±0.007	0.3184 ±0.003	0.3976 ±0.016	0.4320 ±0.007	0.0923 ±0.015	0.1914 ±0.011	0.6596 ±0.007	0.2817 ±0.021	0.4053 ±0.018	0.3997 ±0.013
cosine	**0.2379 ±0.012**	**0.4029 ±0.019**	**0.4035 ±0.008**	**0.3361 ±0.018**	**0.4606 ±0.017**	**0.2716 ±0.009**	**0.1573 ±0.011**	**0.3037 ±0.016**	**0.2434 ±0.010**	**0.3264 ±0.014**	**0.4811 ±0.017**	**0.3798 ±0.011**

Distance Metric Module. We conducted an ablation study on distance metrics in contrastive learning and compared the performance of three different distance metrics—L1 norm, L2 norm, and cosine similarity—across four datasets (see Table S8). On the Her2ST dataset, the cosine similarity method performed the best across all evaluation metrics, with an average PCC (M) of 0.2379, an average PCC (H) of 0.4029, and an average MSE of 0.4035. Similarly, on the other three datasets, the cosine similarity norm method also demonstrated superior performance, highlighting its advantage in distance metrics. These results suggest that, as a distance metric, cosine similarity is better at capturing relationships between gene expressions, thereby improving the accuracy of gene expression prediction.

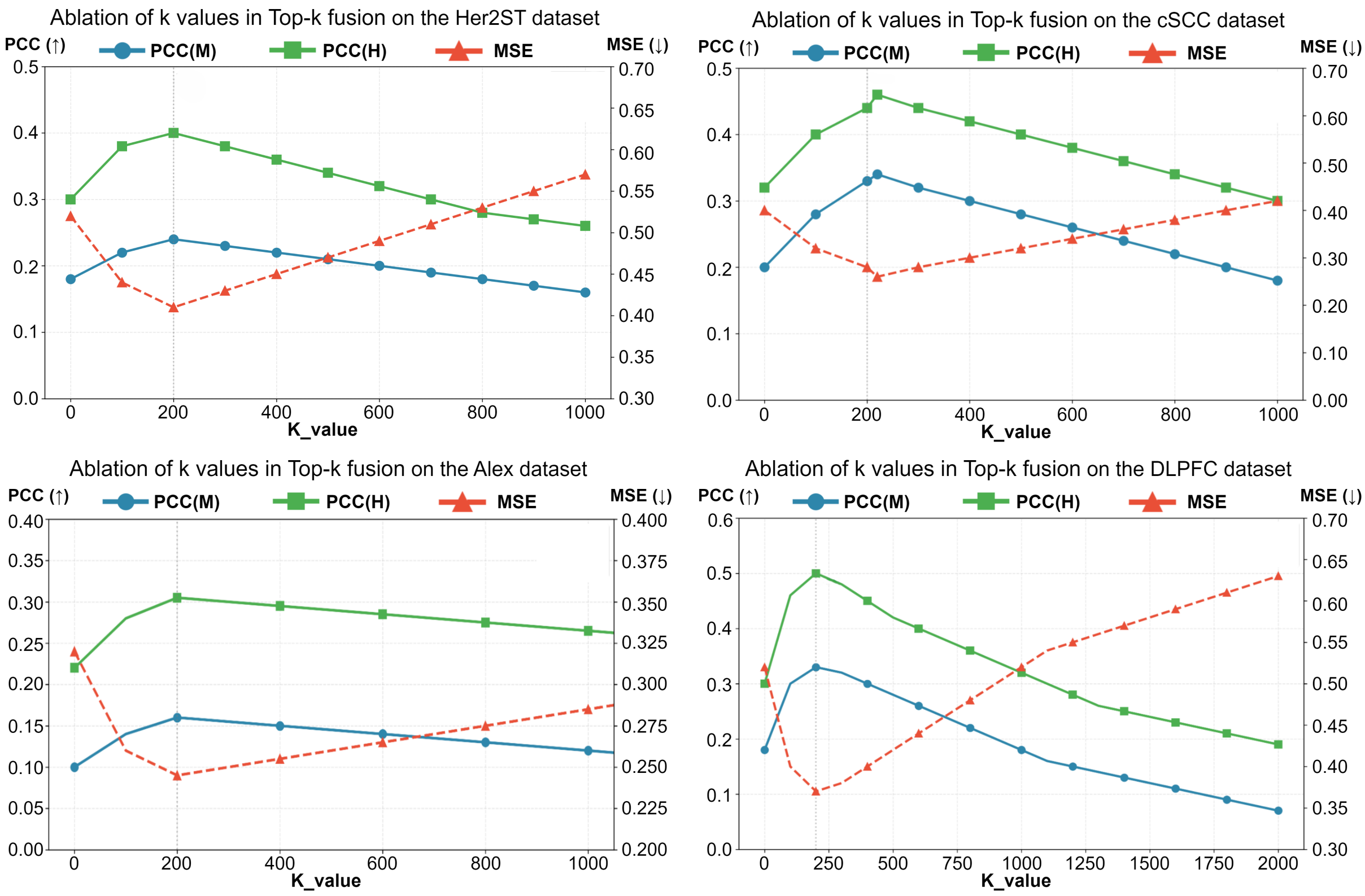

Fig. S4: Sensitivity analysis of hyperparameter k on the Her2ST, cSCC, Alex, and DLPFC datasets. The results of PCC(M), PCC(H), and MSE across different k values show that the model achieves stable and optimal performance around k=200.

Top-k Fusion Hyperparameter k. We conducted an ablation study on the hyperparameter k in the Top-k similar spots weighted fusion strategy during the inference stage and compared the performance under different k values across four datasets (see Fig. S4). On the Her2ST, cSCC, and Alex datasets, the model achieved stable and optimal performance across all evaluation metrics when k was set to

200. Similarly, on the DLPFC dataset, the model also showed the best overall performance around k=200.Across all datasets, the proposed model consistently obtained the highest PCC (M), PCC (H), and lowest MSE at k≈200. These results demonstrate that the selected k value is robust and effective in fusing information from similar spatial spots, which helps improve the stability and reproducibility of spatial gene expression prediction.

S9 Gene-wise SSIM Results

To further quantitatively evaluate the spatial structural consistency of the predicted gene expression patterns, SSIM was introduced as an additional quantitative evaluation metric. We selected the top 50 highly variable genes with the highest expression levels, and calculated gene-wise SSIM across all datasets. Specifically, for each gene, SSIM was calculated slice by slice between the predicted and ground-truth spatial expression maps, and the average value across all slices was taken as the final gene-level score.

We present the gene-wise SSIM distribution of all comparison methods on four datasets, including Her2ST, cSCC, Alex and DLPFC. Fig. S5 shows that our method achieves higher median SSIM and a more compact distribution than baseline models in all datasets. This demonstrates that our model can effectively preserve the spatial structure and continuity of gene expression patterns, and generate prediction results with superior spatial coherence and biological rationality.

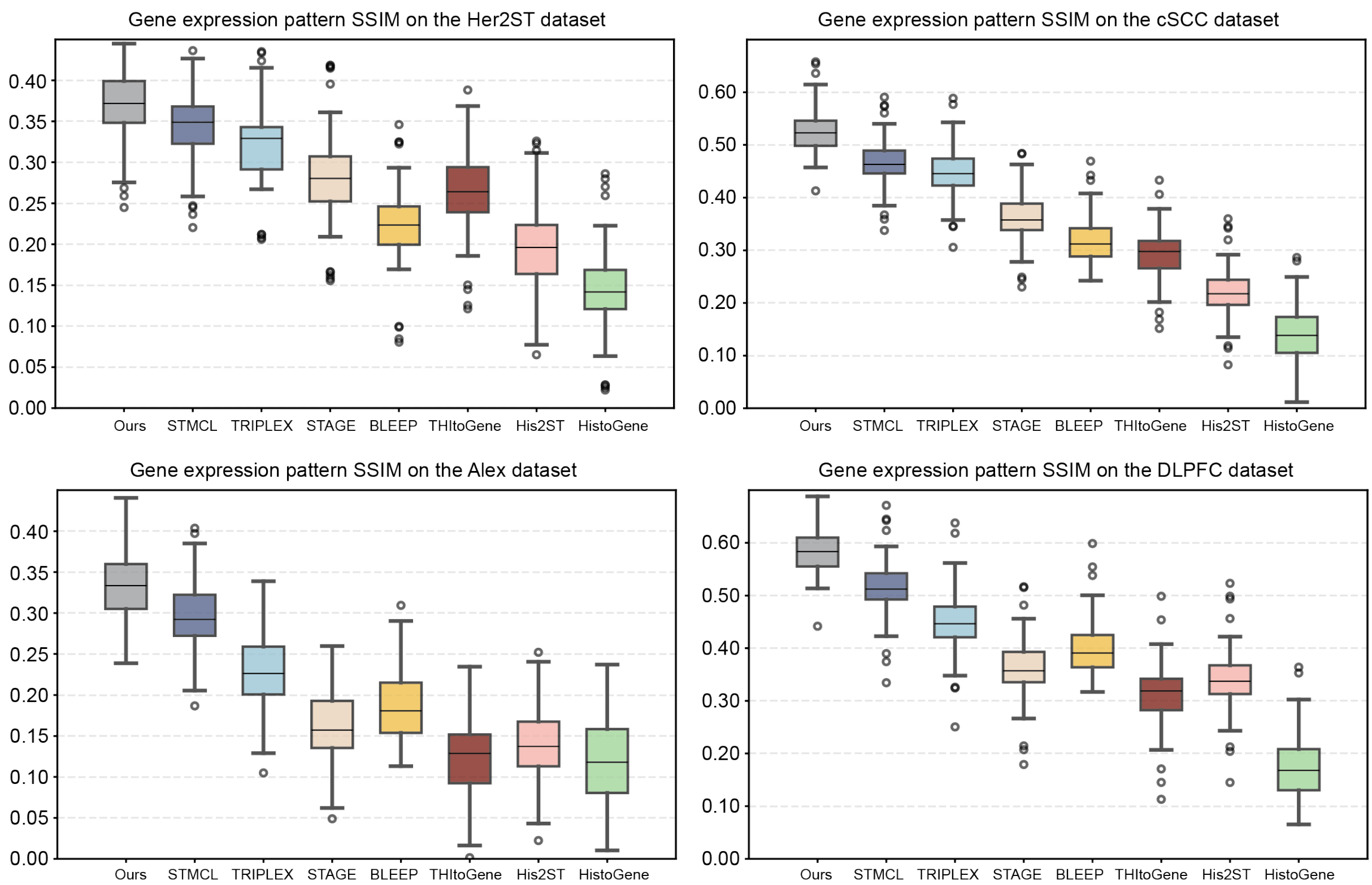

Fig. S5: Comparison of gene expression pattern SSIM across datasets. Boxplots show the distribution of gene-wise SSIM values between predicted and ground-truth spatial gene expression maps for different methods on the Her2ST, cSCC, Alex, and DLPFC datasets.

References

Andersson, A., Larsson, L., Stenbeck, L., et al. (2020). Spatial deconvolution of HER2-positive breast tumors reveals novel intercellular relationships. *BioRxiv*, pages 2020–07.

Chen, R. J., Ding, T., Lu, M. Y., Williamson, D. F., Jaume, G., Song, A. H., Chen, B., Zhang, A., Shao, D., Shaban, M., et al. (2024). Towards a general-purpose foundation model for computational pathology. *Nature Medicine*, 30(3):850–862.

Chung, Y., Ha, J. H., Im, K. C., and Lee, J. S. (2024). Accurate spatial gene expression prediction by integrating multi-resolution features. In *Proceedings of the IEEE/CVF Conference on Computer Vision and Pattern Recognition*, pages 11591–11600.

Ding, T., Wagner, S. J., Song, A. H., Chen, R. J., Lu, M. Y., Zhang, A., Vaidya, A. J., Jaume, G., Shaban, M., Kim, A., et al. (2025). A multimodal whole-slide foundation model for pathology. *Nature Medicine*, pages 1–13.

He, B., Bergenstråhle, L., Stenbeck, L., Abid, A., Andersson, A., Borg, Å., Maaskola, J., Lundeberg, J., and Zou, J. (2020). Integrating spatial gene expression and breast tumour morphology via deep learning. *Nature Biomedical Engineering*, 4(8):827–834.

He, K., Zhang, X., Ren, S., and Sun, J. (2016). Deep residual learning for image recognition. In *Proceedings of the IEEE Conference on Computer Vision and Pattern Recognition*, pages 770–778.

Janesick, A., Shelansky, R., Gottscho, A. D., et al. (2023). High resolution mapping of the tumor microenvironment using integrated single-cell, spatial and in situ analysis. *Nature Communications*, 14(1):8353.

Ji, A. L., Rubin, A. J., Thrane, K., Jiang, S., Reynolds, D. L., Meyers, R. M., Guo, M. G., George, B. M., Mollbrink, A., Bergenstråhle, J., et al. (2020). Multimodal analysis of composition and spatial architecture in human squamous cell carcinoma. *Cell*, 182(2):497–514.

Jia, Y., Liu, J., Chen, L., Zhao, T., and Wang, Y. (2024). THItoGene: a deep learning method for predicting spatial transcriptomics from histological images. *Briefings in Bioinformatics*, 25(1):464–474.

Laha, D., Grant, R., Mishra, P., and Nilubol, N. (2021). The role of tumor necrosis factor in manipulating the immunological response of tumor microenvironment. *Frontiers in Immunology*, 12:656908.

Li, S., Gai, K., Dong, K., Zhang, Y., and Zhang, S. (2024). High-density generation of spatial transcriptomics with stage. *Nucleic Acids Research*, 52(9):4843–4856.

López-Urrutia, E., Campos-Parra, A., Herrera, L. A., and Pérez-Plasencia, C. (2017). Alternative splicing regulation in tumor necrosis factor-mediated inflammation. *Oncology Letters*, 14(5):5114–5120.

Lu, M. Y., Chen, B., Williamson, D. F., Chen, R. J., Liang, I., Ding, T., Jaume, G., Odintsov, I., Le, L. P., Gerber, G., et al. (2024). A visual-language foundation model for computational pathology. *Nature Medicine*, 30(3):863–874.

Maynard, K. R., Collado-Torres, L., Weber, L. M., Uytingco, C., Barry, B. K., Williams, S. R., Catallini, J. L., Tran, M. N., Besich, Z., Tippani, M., et al. (2021). Transcriptome-scale spatial gene expression in the human dorsolateral prefrontal cortex. *Nature Neuroscience*, 24(3):425–436.

Pang, M., Su, K., and Li, M. (2021). Leveraging information in spatial transcriptomics to predict super-resolution gene expression from histology images in tumors. *BioRxiv*, pages 1–31.

Shi, Z., Zhu, F., and Min, W. (2025). Inferring multi-slice spatially resolved gene expression from H&E-stained histology images with STMCL. *Methods*, 234:187–195.

Xie, R., Pang, K., Chung, S., Perciani, C., MacParland, S., Wang, B., and Bader, G. (2023). Spatially resolved gene expression prediction from histology images via bi-modal contrastive learning. *Advances in Neural Information Processing Systems*, 36:70626–70637.

Zeng, Y., Wei, Z., Yu, W., Yin, R., Yuan, Y., Li, B., Tang, Z., Lu, Y., and Yang, Y. (2022). Spatial transcriptomics prediction from histology jointly through transformer and graph neural networks. *Briefings in Bioinformatics*, 23(5).
